# Engineered RNA-based activation system for coronavirus sensing in live cells

**DOI:** 10.1016/j.bidere.2025.100040

**Published:** 2025-07-17

**Authors:** Leiping Zeng, Christian Otero, Lei S. Qi

**Affiliations:** a Department of Bioengineering, Stanford University, Stanford, CA, 94305, USA; b Department of Chemical Engineering, Stanford University, Stanford, CA, 94305, USA; c Sarafan ChEM-H, Stanford University, Stanford, CA, 94305, USA; d Chan Zuckerberg Biohub – San Francisco, San Francisco, CA, 94158, USA

**Keywords:** RNA sensor, Coronavirus, Live cell detection, Antiviral, Synthetic biology

## Abstract

Real-time sensing of viral infection in live cells is crucial for virology research and antiviral development. However, existing methods face challenges of low signal sensitivity and the necessity for viral manipulation and cell fixation. Here, we develop a Viral-Engineered RNA-based Activation System (VERAS) that harnesses the viral replicase to induce transgene expression upon viral infection. VERAS is designed to detect real-time viral transcription and replication in live cells, which can trigger the translation of reporter and therapeutic genes. By integrating a viral packaging sequence, VERAS can also be transmitted to neighboring cells through progeny virions, effectively acting as a ‘Trojan Horse’. The negative-stranded VERAS elements demonstrated effective detection of several coronaviruses, including 229E and OC43, due to the conservation of cis-acting RNA structures across coronaviruses. Notably, VERAS functions as a dual-purpose system, acting both as an infection detector and inducible antiviral system. VERAS has the potential for basic virology research applications and can be adopted in improving the inducible expression of mRNA medicines for future coronaviruses.

## 1 Introduction

Coronaviruses are a diverse family of positive-sense, single-stranded RNA viruses that include strains causing the common cold, such as 229E and OC43, and severe acute respiratory syndrome coronavirus 2 (SARS-CoV-2), the causative agent of the COVID-19 pandemic [1]. The continual emergence of novel strains and their rapid viral evolution remains a concern, and continued monitoring and therapeutic development are essential to mitigate the threat posed by evolving coronaviruses.

Real-time methods for continuously sensing and responding to viral infection and replication offer powerful tools for advancing research, diagnostics, and antiviral strategies [[2], [3], [4], [5], [6], [7], [8], [9], [10], [11], [12], [13]]. These approaches enable precise detection and targeting of infected cells, thereby improving our understanding of viral biology and supporting therapeutic development. Despite progress in live-cell biosensing using methods such as RNA toehold sensors [2], FlipGFP [3,4,[7], [8], [9]], luminescent protease reporters [5,9,10], and ADAR-based RNA sensors [6,11,12], current methods are hindered by technical limitations. These include high background noise, suboptimal sensitivity, or reliance on destructive readouts that preclude continuous monitoring. This inability to accurately capture real-time viral replication dynamics within intact cells represents a significant technological gap.

**Fig. 1:**
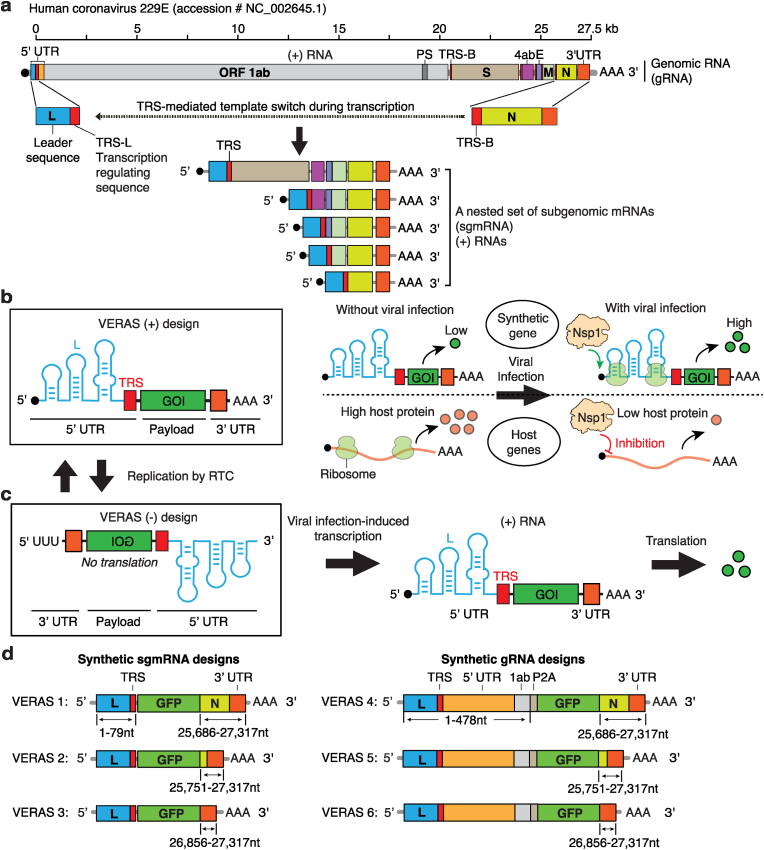
**The design of coronavirus VERAS RNAs. (a)** A scheme depicting the structure of genomic and subgenomic RNAs of 229E and the viral RNA's discontinuous synthesis strategy. **(b)** Design of the positive-stranded VERAS (+) and proposed activities of VERAS-regulated synthetic genes and host genes, with and without viral infection. **(c)** Design of the negative stranded VERAS (−) and proposed activation of transgene expression upon viral infection. **(d)** Design of the VERAS by mimicking the viral genomic and subgenomic RNAs.

To address this gap, we drew inspiration from key coronavirus molecular mechanisms (Fig. 1a). Crucially, the viral Replication-and-Transcription Complex (RTC) specifically recognizes positive-stranded and negative-stranded genomic (gRNA) and subgenomic mRNAs (sgmRNAs), employing a template switch to add a protective 5′ leader sequence during viral mRNA synthesis [1,[14], [15], [16]]. This leader sequence protects viral mRNAs from viral nonstructural protein 1 (Nsp1)-induced endonucleolytic cleavage and translation suppression of host mRNAs, providing an effective strategy for the rapid accumulation of viral RNAs and proteins during infection [17,18]. Furthermore, naturally occurring Defective Interfering (DI) virus particles, despite genomic deletions, retain the RTC-recognized 5′ and 3’ untranslated regions (UTRs) and packaging signals, enabling them to hijack replication machinery and structure proteins from co-infecting viruses for their own replication and DI virus particle assembly [[19], [20], [21], [22], [23]]. This interplay between DI and competent viruses offers a blueprint for engineering synthetic biology systems that exploit viral replication in real-time.

Leveraging these insights, we designed a Viral-Engineered RNA-based Activation System (VERAS), a synthetic RNA element that repurposes native viral biology for conditional gene expression control. Built by fusing engineered coronavirus-derived 5′ and 3′ UTRs to a transgene (e.g., fluorescent reporter or antiviral effector), VERAS acts as a biosensor activated specifically during coronavirus infection, responding sensitively to the viral Nsp1 and RTC activity. This system transforms viral infection into a trigger for synthetic gene regulation, enabling precise spatiotemporal control of diagnostic or therapeutic payloads during active coronavirus replication. VERAS not only enables real-time visualization of viral dynamics but also propagates antiviral signals across cell populations when combined with viral packaging signals. We demonstrate sensitive detection of diverse coronaviruses and dual functionality using VERAS: simultaneous infection monitoring via GFP and therapeutic intervention through interferon expression. This integrated sense-and-respond capability positions VERAS as a versatile tool for studying viral pathogenesis, advancing point-of-care diagnostics, and improving the specificity of RNA-based therapeutics.

## 2 Methods

### 2.1 Cell cultures, viruses, and plasmid design

Human embryonic kidney (HEK293T, Cat# CRL-3216), MRC-5 (Cat# CCL-171), and Vero E6 (Cat# CRL-1586) cells were purchased from ATCC and cultured in 10 ​% fetal bovine serum (Alstem # FB500) in DMEM (Gibco # 10569044). A HEK293T cell line stably expressing the human aminopeptidase N (APN) was made by lentivirus transduction and a monoclonal cell line (termed 293T/hAPN) was selected for infection assays by human coronavirus 229E and OC43. The cell line was named 293T/hAPN. A HEK293T cell line stably expressing the human angiotensin-converting enzyme 2 (ACE2) was made using the same method and named 293T/hACE2. All cell lines were incubated at 37 ​°C in a 5 ​% CO_2_ atmosphere.

Human coronavirus 229E (Cat# VR-740, ATCC) and OC43 (Cat# VR-1558, ATCC) were amplified and titered using MRC-5 ​cells as described previously [24]. All experiments involving the human coronavirus 229E and OC43 were performed in a Biosafety Level (BSL) 2 laboratory. All experiments involving the SARS-CoV-2 were performed in the BSL3 facility at Stanford University.

The hAPN plasmid was purchased from Genscript (Cat#OHu16562C). ACE2 was obtained from Addgene #1786). All of the VERAS expression plasmids used in this study were cloned by following the standard protocol of In-Fusion HD (Takara). The complete VERAS sequences are detailed in the Supplementary Information. For the amplification of the viral untranslated regions (UTRs) and packaging sequences, we utilized reverse transcription polymerase chain reaction (RT-PCR), employing viral RNA as the template. Additionally, reporter genes such as GFP, mRuby, and NanoLuc were obtained from our laboratory's existing plasmid resources. The coding sequences for genes of interest, including Bax, Caspase 3, IFNA4, IFNA5, IFNA8, and IFNA14, were synthesized by Integrated DNA Technologies (IDT).

### 2.2 Quantitative reverse transcription PCR (RT-qPCR)

As described previously [24], the virus titer was quantified using RT-qPCR with the primer pair (5′- CAGTCAAATGGGCTGATGCA-3′ and 5′- AAAGGGCTATAAAGAGAATAAGGTATTCT-3′) and probe (5′-FAM-CCCTGACGACCACGTTGTGGTTCA-BHQ-1-3′). The RNA standard was synthesized with the TranscriptAid T7 High Yield Transcription Kit (Thermo Scientific, Cat# K0441), utilizing a DNA template derived from 229E viral RNA through RT-PCR with primers 5′-TAATACGACTCACTATAGGGAGGTTCCGACGTGCTCGAACTTT-3′ and 5′-AAAAGGGCTATAAAGAGAATAAGGTATTCT-3'. For each sample, total RNA was isolated using a Quick-RNA Viral kit (Zymo Research Cat# R1035). RT-qPCR was performed using the GoTaq Probe 1-Step RT-qPCR System (Promega Cat# A6120).

### 2.3 In vitro transcription (IVT)

The IVT DNA templates were generated by PCR from the VERAS expression plasmids, adding a T7 promoter either to the 5′ terminal for positive strand VERAS or to the 3′ terminal for negative strand VERAS. The VERAS RNAs were synthesized using TranscriptAid T7 High Yield Transcription Kit (Thermo Scientific Cat# K0441). The template DNA was eliminated from the reaction using DNase I. The positive sense RNAs were capped with vaccinal capping enzyme (NEB #M2080S) and methyltransferase (NEB# M0366S) by following the vendor's manuscripts.

### 2.4 Transient transfection and VERAS activity tests

293T/hAPN cells were seeded at 100 k per well in a 24-well plate one day in advance. A transfection mix was prepared for each condition by adding a total of 500 ​ng of plasmid to 50 ​μL of Opti-MEM (Gibco). 1.5 ​μL of TransIT-LT1 transfection reagent was then added and the entire transfection mixed through vigorous pipetting. The transfection mix was incubated for 30 ​min before being added dropwise to cells. For assays including a nuclear BFP transfection marker, a plasmid ratio of 80 ​% VERAS and 20 ​% transfection marker was used. After 3 ​h, the cells were inoculated with 229E, OC43, or SARS-CoV-2 at an MOI of 1.0. For GFP-encoding VERAS assays, the cells were scanned for GFP expression using the IncuCyte (Sartorius) live cell imaging system for 3 days. GFP intensity time course data was calculated by using the IncuCyte analysis software. For Nano luciferase-encoding VERAS assays, cells were lysed, and luciferase activity was measured using a BioTek plate reader according to the manufacturer's instructions for the Nano-Glo® Luciferase Assay System (Promega #N1110).

### 2.5 dsRNA and 229E viral antigen co-staining and immunofluorescence

293T/hAPN cells were seeded at a density of 75k cells/well in a μ-Slide 8 Well Glass Bottom (Ibidi #80827) chambered coverslip pre-coated with 35 ​μg/ml of poly-D-lysine (Gibco). The next day, cells were transfected with VERAS plasmids alongside a nuclear BFP transfection marker. 229E virus (MOI ​= ​1) was added 3 ​h post-transfection.

48 ​h post infection, cells were fixed in 4 ​% paraformaldehyde for 20 ​min. Cells were washed at least 3 times in DPBS (Gibco) and stored at 4C until staining. Cells were blocked for 1 ​h at RT in blocking buffer (BB) consisting of 3 ​% BSA (Santa Cruz Biotechnologies) ​+ ​0.5 ​% Triton X-100 (Sigma-Aldrich) in DPBS. Cells were stained overnight at 4 ​°C with mouse J2 anti-dsRNA (SCICONS #10010200) and rabbit anti-HCoV 229E spike (The Native Antigen Company, #PAB21477–500) primary antibodies diluted 1:250 in BB. Cells were washed 3x in wash buffer (WB) consisting of 0.2 ​% BSA ​+ ​0.1 ​% Triton-X in DPBS to remove excess antibody. Donkey anti-mouse CF568 (Sigma-Aldrich, SAB4600075) and donkey anti-rabbit AlexaFluor 647 (Invitrogen, A-31573) secondary antibodies were diluted 1:500 in BB and added to cells for at least 30 ​min at room temperature. Cells were washed in 3x with WB and once in DPBS to remove residual secondary antibody.

NucBlue™ Fixed Cell ReadyProbes™ Reagent (DAPI) (Invitrogen, #R37606) was added to untransfected cells to visualize nuclei. Cells were imaged in DPBS with a Leica DMI8 inverted microscope equipped with the 63x oil objective, a Leica DFC9000 CT camera, and a Lumoncor SOLA SM II 405 light engine. Images were processed using Fiji/ImageJ and analyzed in CellProfiler to quantify intensities and compute correlations.

### 2.6 Flow cytometry

At assay endpoints, 293T/hAPN cells were washed with 1x DPBS to remove debris and detached using 0.05 ​% Trypsin-EDTA (Gibco). Cells were centrifuged at 400×*g* for 4 ​min and resuspended in FACS buffer (2 ​% FBS, 1 ​mM EDTA in DPBS) before being passed through a cell strainer to obtain a single cell suspension. Flow cytometry was performed using a Cytoflex S Cytometer (Beckman-Coulter). Data was analyzed using FlowJo 10.10.0 and Python. The gating strategies are detailed in Supplementary Fig. 1. Briefly, events were first gated for live, single cells (Supplementary Figs. 1d, f, g). For experiments using a nuclear tagBFP transfection marker, transfected cells were gated using the 99.995th percentile of tagBFP expression in untransfected cells (Supplementary Fig. 1g). GFP and mRuby3 positivity was determined as the 99th percentile of respective expression levels for cells transfected with the tagBFP marker only.

### 2.7 VERAS RNA transcript detection

As described previously [25], the T7 RNA polymerase will generate 1 ​% reverse transcript. To prove the VERAS RNA is transcribed by virus replicase, an RT-PCR method was performed as described previously [25]. The VERASs were transfected into 293T/hAPN cells and infected with 229E. The total RNA was isolated at 24 hpi and decapped with mRNA decapping enzyme (NEB# M0608S) and ligated head to tail by using T4 RNA ligase 1. Then RT-PCR with the indicated primers (P1 primer (i.e., RT primer 2): 5′-GGGATCACTCTCGGCATGG-3’; P2 primer (i.e., RT primer 1): 5′-AGACACAAAGTCTAAAAAGCAACT-3′) was used to testify whether the anti-sense strand of the VERAS RNA is generated.

### 2.8 VERAS RNA replication detection

The VERAS RNA was prepared by IVT as described previously. 293T/hAPN cells were seeded one day in advance at 200 k per well in a 24-well plate and transfected with 500 ​ng of IVT RNA per well using Lipofectamine MessengerMAX (Thermo # LMRNA003). After 1 ​h, the cells were infected with 229E at an MOI of 1.0 and were lysed with RNA lysis buffer (Zymo# R1035) at 1, 10, 22, and 36 hpi. Total RNA was isolated and the VERAS RNA was quantified using RT-qPCR with a primer pair (5′-ACGACGGCAACTACAAGACC-3′ and 5′-TTGTACTCCAGCTTGTGCCC-3′). RT-qPCR was performed using the iScript™ cDNA Synthesis Kit and iTaq Universal SYBR Green Supermix (Bio-Rad) and run on a Biorad CFX384 real-time system (C1000 Touch Thermal Cycler), according to the manufacturer's instructions. The relative VERAS RNA abundance was normalized to the *GAPDH* internal control which was quantified with a primer pair (5′-GTCTCCTCTGACTTCAACAGCG-3′ and 5′-ACCACCCTGTTGCTGTAGCCAA-3′). The relative RNA abundance of each timepoint was normalized to the 1 hpi samples.

### 2.9 Progeny virion package test

The 293T/hAPN cells were transfected with VERAS expressing plasmid and inoculated with 229E or OC43 at an MOI of 0.1. At 48 hpi, the supernatant was collected by spinning at 4000 ​g for 5 ​min and filtering through 0.45 ​μm and used to inoculate fresh 293T/hAPN cells. GFP expression was measured using the IncuCyte system for 2 days.

### 2.10 Co-culture assay

The GFP^+^ cells were first prepared by transfecting 293T/hAPN cells with a GFP-expressing construct. A separate group of 293T/hAPN cells were either not transfected or transfected with positive or negative strand VERAS encoding IFNA8 and mixed with the GFP^+^ cells at a ratio of 1:2 or 1:4. The cells were challenged with 229E at an MOI of 0.1. The Cytotox red dye (Sartorius # 4632) for counting dead cells was added to the media at a final concentration of 100 ​nM. The dead cells were counted using the IncuCyte system for 3 days.

### 2.11 Data analysis

Data visualizations (graphs) were performed in GraphPad Prism software version 9 and Python 3.9.7. P values were calculated in Excel and GraphPad Prism. Statistical analyses were performed using a two-sided *t*-test with equal variance for all data except that one-sided *t*-test with equal variance was used for the data of Fig. 4, Fig. 5f, and Supplementary Fig. 4e–f p values for all figures are provided in the Source data. A p value ​< ​0.05 is considered statistically significant.

## 3 Results

### 3.1 Design of a synthetic RNA sensor for coronavirus transcription detection

Coronaviruses synthesize genomic RNA (gRNA) and subgenomic mRNAs (sgmRNAs) in a nested, discontinuous manner, involving the synthesis of negative viral RNA strands as the templates for the positive strand gRNA and sgmRNAs [14]. Furthermore, nonstructural protein 1 (Nsp1) regulates viral versus host mRNA translation by dual mechanisms: it induces endonucleolytic cleavage of host capped mRNAs and inhibits translation initiation by binding to the ribosomal 40S subunit, thereby blocking the mRNA entry tunnel [17,18]. However, the leader sequence (L) in the 5’ UTR of viral mRNAs counteracts Nsp1-mediated suppression, ensuring preferential translation of viral transcripts [18].

Building on these observations, we designed a dual-mechanism system for conditional transgene expression. First, the system leverages a coronavirus 5’ UTR leader sequence to protect viral mRNA translation during infection (Fig. 1b). Second, the negative-stranded VERAS constructs rely on the RTC to first convert them into positive-sense mRNAs, a critical step for transgene expression (Fig. 1c). Additionally, the VERAS constructs may be replicated by viral RTC through bidirectional conversion between negative-sense strands to complementary positive-sense strands (Fig. 1b–c). We hypothesized that these features could be harnessed as a mechanism for conditional expression of desired reporter and therapeutic genes, dependent on the infection status of the cell (Fig. 1b–c).

While the composition of the 5′ UTR, including the leader sequence and TRS, is determined for effective replication, transcription, and translation of the viral gRNA and sgmRNAs, the minimal essential sequences of the 3’ termini remain uncharacterized [26]. To develop RNA sensors responsive to coronavirus infection, we repurposed regulatory elements from the human coronavirus 229E. We designed six virus-activated RNA variants (VERAS 1–6), with three mimicking the shortest viral sgmRNA (VERAS 1–3) and three emulating the viral gRNA (VERAS 4–6) (Fig. 1d).

**Fig. 2:**
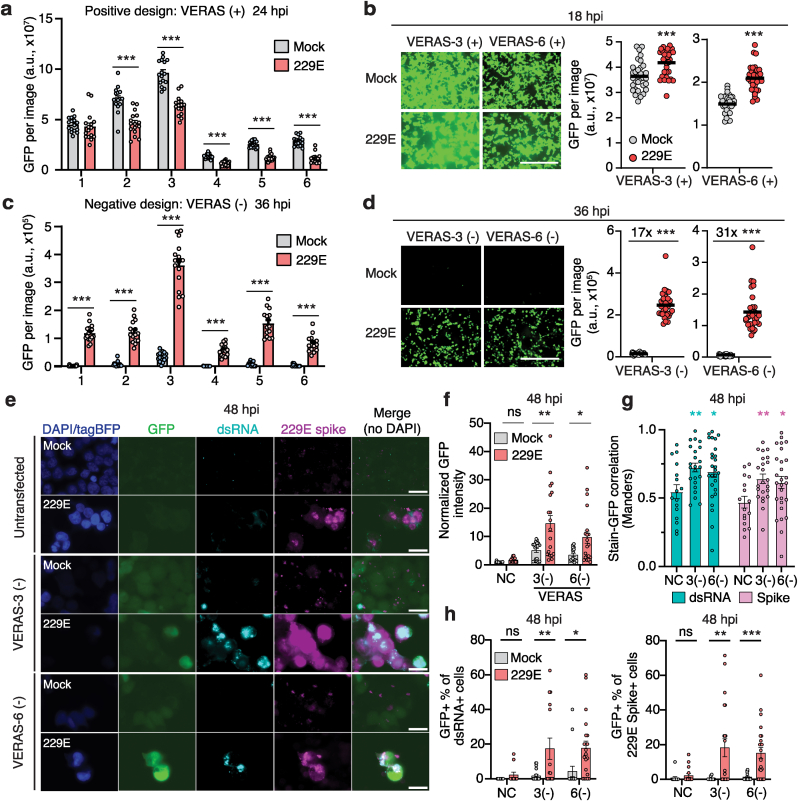
**Coronavirus infection induces VERAS-mediated reporter expression. (a**–**d)** GFP expression from VERAS (+) **(a**–**b)** and VERAS (−) **(c**–**d)** with and without 229E infection. GFP intensity is the integrated signal measured using the IncuCyte Live-cell Imaging System. 4 independent biological replicates were performed (4 images were taken for **a** and **c**, and 8 images for **b** and **d** per biological replicate). Bar represents mean of each group, and each dot represents an individual image. Scale bar in (**b**) and (**d**), 400 ​μm. (**e**) Immunofluorescence imaging of 293T/hAPN cells transfected with VERASs and infected with 229E. TagBFP marks transfected cells; dsRNA and spike protein co-stained. 3 independent biological replicates were performed, with 8–12 images taken per replicate. Scale bar, 20 ​μm. (**f**) GFP intensity in VERAS transfected cells, comparing 229E vs. mock infected conditions. (**g**) Mander's correlation coefficients between dsRNA or 229E spike vs. GFP signal in cells infected with 229E comparing transfection with VERASs and untransfected control. (**h**) GFP percent positivity of cells stained positive for 229E spike or dsRNA, comparing 229E vs. mock infected conditions. Data are presented as mean ​± ​s.e.m. P values were calculated by two-tailed Student's t tests. n.s., not significant; ∗, P ​< ​0.05; ∗∗, P ​< ​0.01; ∗∗∗, P ​< ​0.001. Source data and P values are provided.

To test the functionality of different designs, we generated a HEK293T cell line (293T/hAPN) stably expressing the human APN (Aminopeptidase N, also known as CD13), an established receptor for human coronavirus 229E. We transfected cells with plasmids encoding different VERAS designs and subsequently infected transfected cells with 229E. To facilitate accurate RNA processing at the 3′ end, all designs were also expressed under a CMV promoter with an incorporated HDV ribozyme (Supplementary Fig. 1a). While none of the positive designs showed 229E-induced reporter expression (Fig. 2a), all negative designs demonstrated a strong induction (Fig. 2c). RNA designs with shorter 3’ UTR (VERAS-3 and 6) displayed better activation upon viral infection.

We further characterized the GFP expression dynamics for both positive and negative designs of VERAS-3 and VERAS-6 and confirmed that the negative designs exhibited robust reporter activation, with 17- and 31-fold increase in reporter expression upon 229E infection at 36 ​h post-infection (hpi) (Fig. 2b and d, Supplementary Fig. 1b–c). Furthermore, flow cytometry confirmed that GFP expression was significantly activated at the single-cell level for both VERAS-3 (−) and VERAS-6 (−) in response to 229E infection (Supplementary Fig. 1d–e). These results demonstrate the functional specificity of the RNA designs in regulating gene expression during viral infection.

A key advantage of VERAS is that it can be used to detect and respond to coronavirus infection in live cells, while traditional methods require fixation or lysis to reliably detect infection. To confirm the specificity of the negative-stranded VERAS-3 and VERAS-6 for detecting 229E infection, we performed immunofluorescence co-staining for double stranded RNA (dsRNA) and 229E spike protein (Fig. 2e), which are established markers of viral infection [27]. We observed a global increase in GFP intensity in cells transfected with VERAS-3(−) and VERAS-6(−) upon 229E infection compared to untransfected controls as previously observed (Fig. 2f). Cells under 229E infection that received VERASs had higher correlation between both dsRNA/Spike and GFP signals, indicating that GFP expression was associated with viral infection (Fig. 2g). Among cells that were stained positive for dsRNA and 229E Spike, VERAS-3(−) and VERAS-6(−) cells also exhibited a significantly higher percentage of GFP ​+ ​cells in the infected populations compared to the uninfected populations (Fig. 2h). These findings confirm the sensitivity of VERAS to detect coronavirus infection, with transgene expression levels correlating with established viral infection markers.

### 3.2 Characterization of VERAS for coronavirus transcription detection

**Fig. 3:**
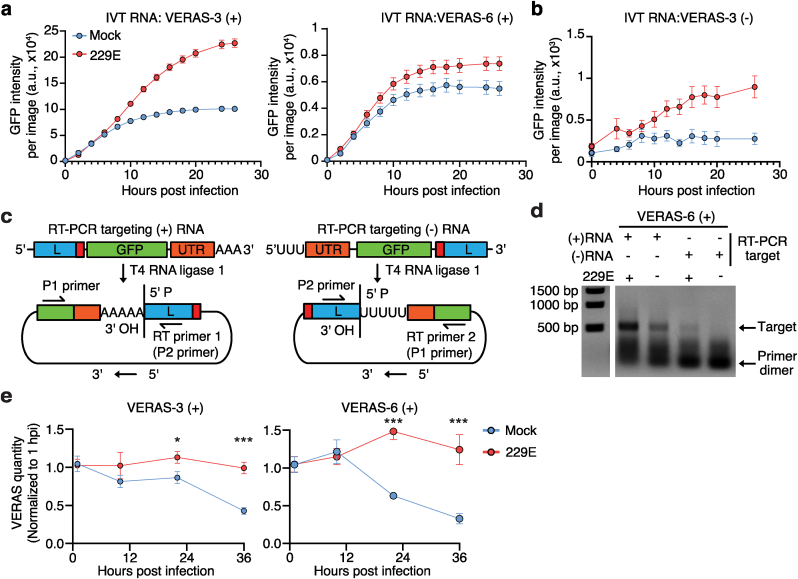
**VERASs are transcribed and replicated during coronavirus infection. (a**–**b)** GFP expression dynamics in 293T/hAPN cells transfected with in vitro transcribed VERAS-3 and -6 (positive strand, **a**) or VERAS-3 (negative strand, **b**) following 229E infection. **(c)** RT-PCR-based detection of (+) and (−) RNA transcripts from VERASs. **(d)** Gel image of RT-PCR products for detection of the (−) RNA transcribed from (+) VERAS at 24 hpi. **(e)** Replication of VERAS-3 (+) and VERAS-6 (+) during 229E infection. Cells were transfected with the in vitro transcribed RNAs, infected with 229E, and total cellular RNA was collected at 1, 10, 22, and 36 hpi. VERAS RNA levels were quantified by RT-qPCR and normalized to 1 hpi. Data represents 3 biological replicates (3 technical repeats each), shown as mean ​± ​s.e.m. Statistical significance determined by two-tailed Student's t-test: n.s., not significant; ∗P ​< ​0.05; ∗∗∗P ​< ​0.001. Source data and P values are provided.

To further elucidate the mechanism of VERAS, we transfected cells with in vitro transcribed (IVT) positive VERASs. Following 229E infection, cells displayed significantly higher GFP reporter expression than the mock-infected cells (Fig. 3a). While the negative-stranded VERAS needs to be transcribed into its corresponding positive strand for protein translation (Fig. 1c), 229E infection induces the reporter expression from the negative VERAS (Fig. 2, Fig. 3b), implying it is transcribed into positive sense RNAs by the viral RTC.

**Fig. 4:**
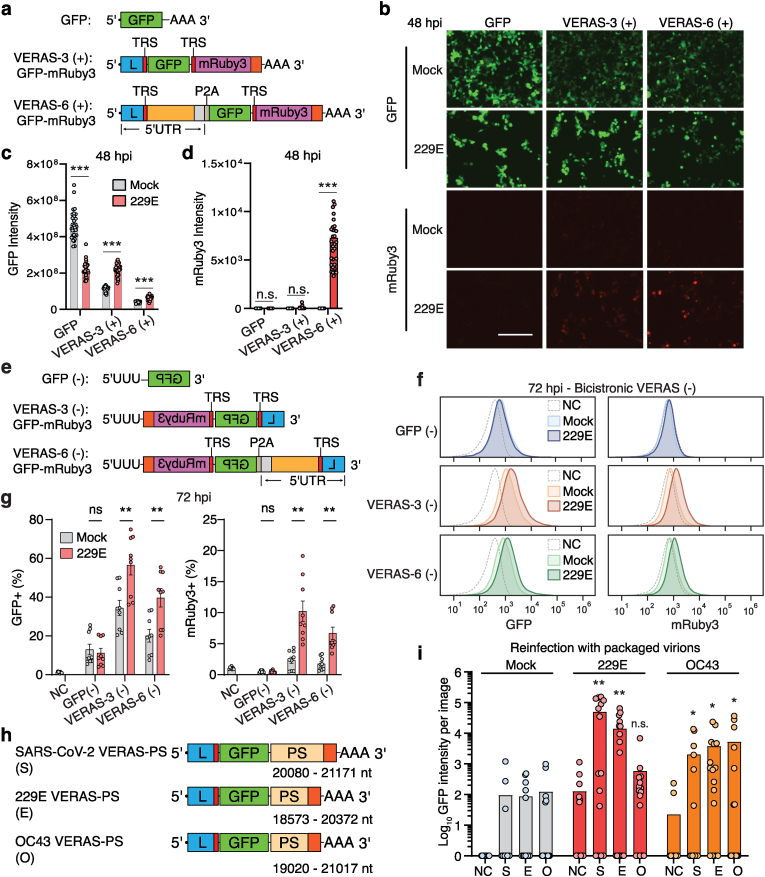
**VERASs encode additional proteins and are packaged into progeny virions for cell transmission. (a)** Positive-strand genomic and subgenomic VERAS designs encoding both GFP and mRuby3. **(b**–**d)** GFP and mRuby3 expression in the 293T/hAPN cells with or without 229E infection at 48 hpi. Bars: means; circles: technical replicates (n ​= ​4 biological replicates, 8 images each). Scale bar, 200 ​μm. **(e)** Negative strand bicistronic VERAS designs. **(f**–**g)** Flow cytometry quantification of GFP and mRuby3 expression from VERAS (−) (*n* ​= ​3 biological replicates, 3 technical replicates each). **(h)** SARS-CoV-2 (S), 229E (E), and OC43 (O) VERASs with packaging sequences. **(i)** GFP signal in cells re-infected with virions from VERAS-transfected (S, E, O) or control (NC) cells, mock vs 229E or OC43 infection. Bars: means; circles: individual images (n ​= ​4 biological replicates, 4 images each). Statistical significance: two-tailed *t*-test (b–d, f–g); one-tailed *t*-test (i); n.s., not significant; ∗P ​< ​0.05; ∗∗P ​< ​0.01; ∗∗∗P ​< ​0.001. Source data available.

**Fig. 5:**
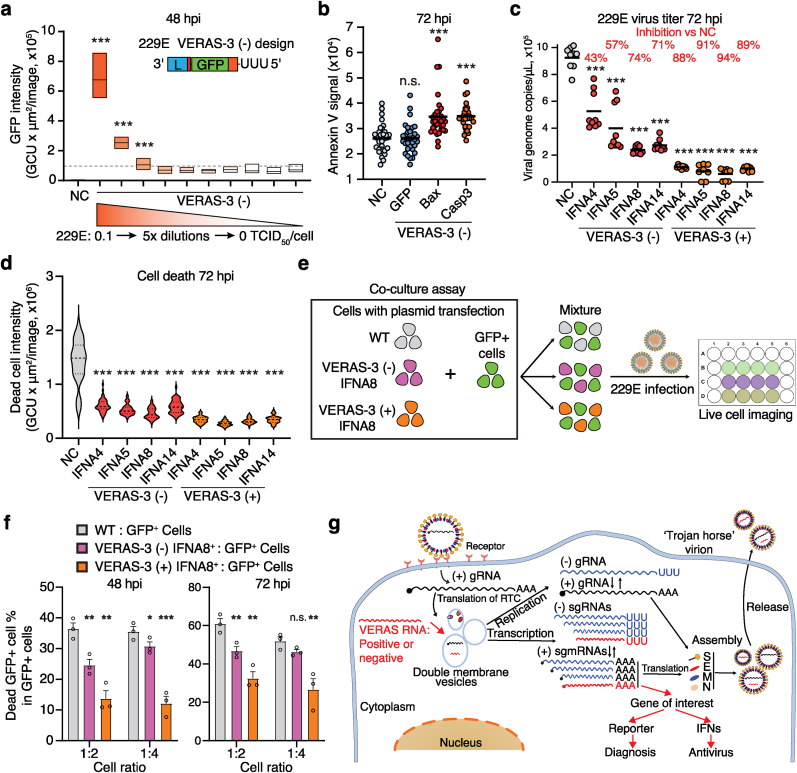
**VERASs for live-cell virus detection and infection-activated antiviral. (a)** GFP intensity in 293T/hAPN cells infected with or without 229E virus at various loads. Data shown as box plots (median, 25–75th percentiles, min–max) from 4 biological replicates (8 technical repeats each). **(b)** Apoptosis signal (Annexin V) in cells transfected with or without VERASs encoding apoptosis inducers (Bax or Caspase3) and infected with 229E. Data from 4 biological (8 images each). **(c)** 229E virus titers in media from cells transfected with or without a VERAS expressing IFNα subtypes (positive or negative strand) and infected with 229E. Data from 3 biological replicates (3 technical repeats each), shown as mean (bar) and individual data points (circles). **(d)** Death signal (Cytotox green dye) of the infected cells. Data from 3 biological replicates (16 images each), presented as violin plots. **(e)** Scheme of co-culture assay to assess protective effects of VERAS-3 (+) or VERAS-3 (−) IFNA8 on neighboring cells. **(f)** Death rate of GFP^+^ cells in total GFP^+^ cells. One-tailed Student's t-tests for p values. **(g)** Mechanism and future applications of VERAS system that harnesses viral regulatory machineries to detect virus infection and trigger antiviral therapy. Data from 3 biological replicates, shown as mean ​± ​s.e.m. Statistical Analysis: P values calculated by two-tailed Student's t-test unless otherwise noted. NC, no transfection negative control; n.s., not significant; ∗P ​< ​0.05; ∗∗P ​< ​0.01; ∗∗∗P ​< ​0.001. Source data provided.

T7 RNA polymerase produces ∼1 ​% of the reverse complementary strand of the IVT RNAs [28], to determine whether the RNA sensors are transcribed following viral infection, we followed a previously established protocol [25] (Fig. 3c). Briefly, total cellular RNA was decapped and ligated head-to-tail by T4 RNA ligase 1. A primer specific for each RNA strand was used for reverse transcription through the head-to-tail sequence, which was then amplified using PCR. As hypothesized, cells transfected with positive VERAS RNA can be transcribed to negative-strand RNA following viral infection (Fig. 3d). Furthermore, we found that the virus infection can replicate the viral VERAS RNAs, maintaining their abundance, while the IVT RNA decreased in mock-infected cells (Fig. 3e).

### 3.3 VERAS can encode bicistronic genes and package into progeny virions

The transcriptional regulation mechanism of coronaviruses allows them to synthesize sgmRNAs from gRNA, guided by the TRS sequence [14]. Building upon this understanding, we designed VERAS to encode a second protein that could be activated during viral infection (Fig. 4a). We incorporated a subgenomic RNA encoding mRuby3 by inserting a second TRS into both the VERAS-3 and VERAS-6 designs, which mimic subgenomic and genomic RNA, respectively. Following transfection of the positive VERASs into 293T/hAPN cells, we observed GFP expression in all conditions regardless of viral infection. However, mRuby3 expression was only detected in cells infected by the 229E coronavirus (Fig. 4b–d, Supplementary Fig. 2a–c). We noted that the genomic RNA design (VERAS-6) expressed significantly higher levels of mRuby3 compared to the subgenomic RNA design (VERAS-3). Moreover, while GFP protein expression was reduced by infection compared to the mock condition at 48 hpi when VERAS (+) encodes solely the GFP (Supplementary Fig. 1b), its expression increased when encoding bicistronic proteins (GFP and mRuby) (Fig. 4c).

We next characterized VERAS with a bicistronic gene cassette using the negative designs (Fig. 4e). We hypothesized that the efficiency of a negative bicistronic VERAS would be lower than the positive designs, as it requires multiple transcription steps by the viral RTC to express both proteins. Flow cytometry 72 ​h post 229E infection showed that cells transfected with VERAS-3(−) and VERAS-6(−) had higher percentages of cells expressing GFP and mRuby3, with increased mean expression levels of both GFP (1.66x and 1.55x, respectively) and mRuby3 (1.77x and 1.61x, respectively) (Fig. 4f–g, Supplementary Fig. 1f–g). Although the fold changes of GFP and mRuby3 induction in response to virus were similar in each of the designs, we found lower leakiness of mRuby3 in the absence of virus compared to GFP (1.75 ​%–2.34 ​% mRuby3+ vs. 20.1 ​%–34.43 ​% GFP+) (Fig. 4g). This finding highlights the higher sensitivity of negative VERASs for encoding bicistronic genes, whereby RTC-mediated transcription of VERAS and subsequent transcription of subgenomic mRNA encoding mRuby3 greatly decreases background signal as a trade-off for lower efficiency.

We further detected the synthesis of the subgenomic RNA from the positive VERASs using reverse transcription PCR (RT-PCR) (Supplementary Fig. 2d). A smaller secondary product was only generated by VERAS-6(+) in the virus-infected sample (Supplementary Fig. 2e). Sanger sequencing confirmed the association of the leader sequence with the mRuby3 coding sequence, supporting the mechanism of bicistronic gene expression orchestrated by the viral RTC (Supplementary Fig. 2f).

Coronavirus genomic RNA is packaged into progeny virions via a packaging sequence (PS) [29,30]. To explore the concept of packaging VERAS-encoded genes into virions, we integrated a validated PS from SARS-CoV-2 into its VERAS-3 design and introduced potential PSs of 229E and OC43 into their respective VERAS-3 designs (Fig. 4h). All three designs demonstrated comparable GFP expression post-transfection (Supplementary Fig. 2g). We then assessed packaging efficiency by infecting cells with 229E or OC43, collecting viral supernatants at 48 hpi, and using these to infect fresh 293T/hAPN cells. GFP expression in reinfected cells revealed that both SARS-CoV-2 and 229E VERAS-3 designs were effectively packaged by 229E and OC43 virions (Fig. 4i). Unexpectedly, OC43 VERAS-3 was not packaged by 229E virions but was effectively incorporated into OC43 progeny virions (Fig. 4i). These results demonstrate that VERASs can be packaged into progeny virions in a PS-dependent manner, with packaging efficiency varying by viral strain.

### 3.4 VERAS can detect and respond to broad coronaviruses

In addition to the 229E VERASs, we also designed 6 negative-strand VERAS RNAs each for OC43 and SARS-CoV-2, using the same engineering strategy (Supplementary Fig. 3a and c). These were tested in MRC-5 and 293T/hACE2 cells, respectively. Among the OC43 VERASs, those with the longest 3’ sequences, which includes the N protein-coding sequence and the 3′ UTR, displayed the most substantial fold of activation in response to virus infection (Supplementary Fig. 3b). However, SARS-CoV-2 VERASs showed only mild induction of reporter expression due to their high baseline expression in uninfected cells (Supplementary Fig. 3d). Interestingly, we found these VERASs capable of cross-activation upon testing with infection of the three different coronaviruses (Supplementary Fig. 3e–h). The 229E infection successfully triggered reporter expression from VERASs of all three viruses (Supplementary Fig. 3e). In contrast, the OC43 infection only activated reporter expression from 229E VERAS-3, OC43 VERAS-3, and SARS-CoV-2 VERAS-3 (Supplementary Fig. 3f). Notably, infection with SARS-CoV-2 did not appear to activate reporter expression from any of the VERAS designs (Supplementary Fig. 3g). The non-specific activation of VERAS by various viruses upon infection may suggest a certain degree of conservation in the cis-elements and the replication machinery of these coronaviruses.

### 3.5 VERAS has potential for coronavirus detection and antiviral responses

The VERAS mechanism exploits the virus replication machinery to drive self-transcription. When expressed as a negative-strand RNA, VERAS is converted into a positive strand, enabling transgene expression upon viral infection. By encoding a reporter gene, VERAS can serve as a sensitive biosensor for virus detection. We demonstrated that 293T/hAPN cells expressing 229E VERAS-3 (−) detected 229E infection at titers exceeding an MOI as low as 0.004 TCID_50_/cell (Fig. 5a). This highlights the high sensitivity of VERASs for monitoring viral infection dynamics.

Beyond virus detection, VERAS can function as an infection-activated antiviral system by encoding apoptosis inducers or interferons (IFN) (Fig. 5b–d, Supplementary Fig. 4a–d). When apoptosis inducers (Bax or Caspase 3) were encoded in the negative-strand VERAS-3, viral infection triggered transgene expression (Supplementary Fig. 4b) and markedly increased cell apoptosis compared to untransfected cells or GFP encoding VERAS-3 controls (Fig. 5b). This demonstrates the potential of VERASs to serve as programmable antiviral tools that activate cellular defense mechanisms in response to infection.

We encoded four interferon alpha subtypes (IFNα 4, 5, 8, and 14), known to effectively suppress SARS-CoV-2 replication [31], into both positive- and negative-strand 229E VERAS-3 constructs (Supplementary Fig. 4c). As expected, cells transfected with positive-strand VERAS-3 constructs effectively inhibited virus replication and reduced cell death compared to untransfected controls (Fig. 5c–d). Virus titers decreased by ∼80 ​% at 48 hpi and ∼90 ​% at 72 hpi (Fig. 5c, Supplementary Fig. 4d), highlighting the potent antiviral activity of these IFNs against 229E. In contrast, negative-strand VERAS-3 constructs, which rely on viral infection for transgene expression, exhibited delayed and less potent effects. Among the four IFNα subtypes, negative-strand VERAS-3 encoding IFNA8 showed the most potent antiviral effect, reducing virus titers by 67 ​% at 48 hpi and 74 ​% at 72 hpi.

Interferons act as ‘alarm’ signals, priming neighboring cells to activate antiviral defenses. To test whether VERAS-3 RNAs encoding IFNs could protect neighboring cells, we performed a co-culture assay (Fig. 5e). We mixed wild-type, negative-strand, or positive-strand VERAS-3 IFNA8-expressing cells with GFP ​+ ​cells in a ratio of 1:2 or 1:4 and infected the cell mixtures with 229E. We measured total cell death and GFP ​+ ​cell death rates at 48 and 72 hpi. Co-cultures of wild-type and GFP ​+ ​cells showed the highest mortality of both total and GFP ​+ ​cells (Fig. 5f, Supplementary Fig. 4e–f). In contrast, cell expressing positive-strand VERAS-3 IFNA8 reduced overall cell death and effectively protected GFP ​+ ​cells from viral destruction. Similarly, negative-strand VERAS-3 IFNA8 provided significant protection for GFP ​+ ​cells. These findings highlight the potential of negative-strand VERASs as a novel responsive antiviral strategy, where transgene expression is triggered exclusively upon viral infection.

## 4 Discussion

VERAS is a novel RNA-based biosensor that exploits the viral RNA synthesis machinery to detect and respond to coronavirus infection in living cells (Fig. 5g). It incorporates conserved viral 5′ and 3′ UTRs, enabling replication and transcription upon infection, which in turn drives transgene translation, when delivered in either positive- or negative-strand formats. Negative-strand VERAS designs depend on viral RTC activity to produce viral mRNA first, thus reducing baseline transgene expression (Fig. 2c and d). In contrast, positive-strand VERAS designs allow direct ribosome access, leading to higher baseline expression without viral infection (Figs. 1b–Fig. 2a–b). This baseline is further illustrated by the contrast between plasmid- and IVT-delivered positive-strand VERASs (Fig. 2a vs. Fig. 3a): plasmid-derived RNA sustains continuous background expression that masks RTC-mediated amplification during infection, whereas IVT RNA degrades in uninfected cells but undergoes specific replication-driven amplification in infected cells (Fig. 3e). Notably, negative-strand VERAS enables real-time, non-destructive monitoring of coronavirus infection with high sensitivity (MOI ≥0.004 TCID_50_/cell, Fig. 5a), offering potential advantages over existing methods [3,5]. This high sensitivity positions VERAS as a useful tool for *in vivo* imaging of coronavirus infection and replication, eliminating the need for reporter gene knock-in to viral genomes and overcoming limitations of traditional reverse genetics.

VERAS offers dual diagnostic and therapeutic capability. Its bicistronic design allows co-expression of a diagnostic reporter (e.g., fluorescent protein) and a therapeutic effector (e.g., interferons) for simultaneous infection tracking and antiviral delivery (Fig. 4a–g). Incorporation of engineered viral packaging signals allows VERAS transcripts to be packaged into progeny virions (Fig. 4i), creating a “Trojan horse” effect that facilitates spread to naïve cells and enables monitoring of viral transmission across cell populations [[2], [3], [4], [5], [6],^10^]. Negative-strand VERAS function as infection-activated antiviral agents, mitigating virus-induced cell death in both infected and neighboring cells (Fig. 5b–f). We also anticipate that VERAS can be combined with RNA medicines to improve its precision and specificity [32].

Characterization of VERAS variants revealed the importance of RNA architecture on performance. Positive-strand VERASs exhibit high replication fidelity (Fig. 3e). confirming RTC recognition of conserved 3′ UTR elements, consistent with native viral genome amplification [14,16]. Comparing subgenomic (VERAS-3) and genomic (VERAS-6) mimics showed that VERAS-6 induced significantly stronger activation (31-fold vs 17-fold, Fig. 2d) and more robust bicistronic transgene expression (Fig. 4c). This enhancement likely results from its genomic 5′ UTR, which improves RTC processivity during positive-strand synthesis and supports efficient TRS-guided subgenomic transcription [14,16]. Furthermore, VERAS exhibits broad coronavirus activity, with species-specific differences in activation and packaging enabling tailored engineering. Cross-activation between 229E- and OC43-derived VERASs reflects conserved RTC recognition motifs, while SARS-CoV-2 displays unique RNA replication features (Supplementary Fig. 3e–g). The selective incorporation of SARS-CoV-2 PS-containing VERASs into 229E/OC43 virions suggests modular packaging compatibility via shared PS secondary structure (Fig. 4i). Conversely, 229E's inability to package OC43-derived VERASs suggests selective nucleocapsid recognition, highlighting opportunities for PS engineering to tailor cell-to-cell transmission.

This work establishes key design principles for synthetic RNA biosensors, notably the coupling of transgene expression to RTC activity and 5′ and 3′ UTRs, which are highly conserved in coronaviruses that maintain essential structural motifs for viral replication and transcription. Although extreme mutations in these UTRs are theoretically possible, they are evolutionarily constrained by their indispensable roles. Newly emerged coronavirus species may possess different 5′ and 3’ UTR sequences, but the underlying replication mechanisms are likely conserved. Therefore, the design principles of VERAS can be adapted to new strains by incorporating their respective UTRs into the current system. Future efforts should prioritize improving sensitivity to low viral titers, reducing baseline expression for SARS-CoV-2 designs, and quantifying and optimizing packaging efficiency to maximize the Trojan horse effect. Beyond coronaviruses, VERAS exemplifies how viral RNA regulatory elements and replication machineries can be harnessed as synthetic tools for virus detection and RNA therapeutics, offering a versatile framework for synthetic biology applications in infectious disease research.

## Author contributions

L.Z. and L.S.Q. conceived the idea and planned the experiments. L.Z. and C.O. designed and cloned the constructs, performed the experiments, and analyzed the data. L.Z., C.O., and L.S.Q. wrote the manuscript. All authors reviewed and approved the manuscript.

## Declaration of competing interest

The authors declare the following financial interests/personal relationships which may be considered as potential competing interests: L.S.Q. is a founder of Epicrispr Biotechnologies and a scientific advisor of Laboratory of Genomic Research. The other authors declare no competing financial interests.
