# Supplementary material for: Engineered RNA-based activation system for coronavirus sensing in live cells
Source: Biodes Res. 2025 Jul 17;7(3):100040. doi: 10.1016/j.bidere.2025.100040 (PMC12710057; doi:10.1016/j.bidere.2025.100040)
Supplement: Multimedia component 1 [file mmc1.docx]

**Supplementary Information for:**

**Engineered RNA-based Activation System for Coronaviruses Sensing in Live Cells**

Leiping Zeng, Christian Otero, Lei Stanley Qi

**Supplementary Figures**


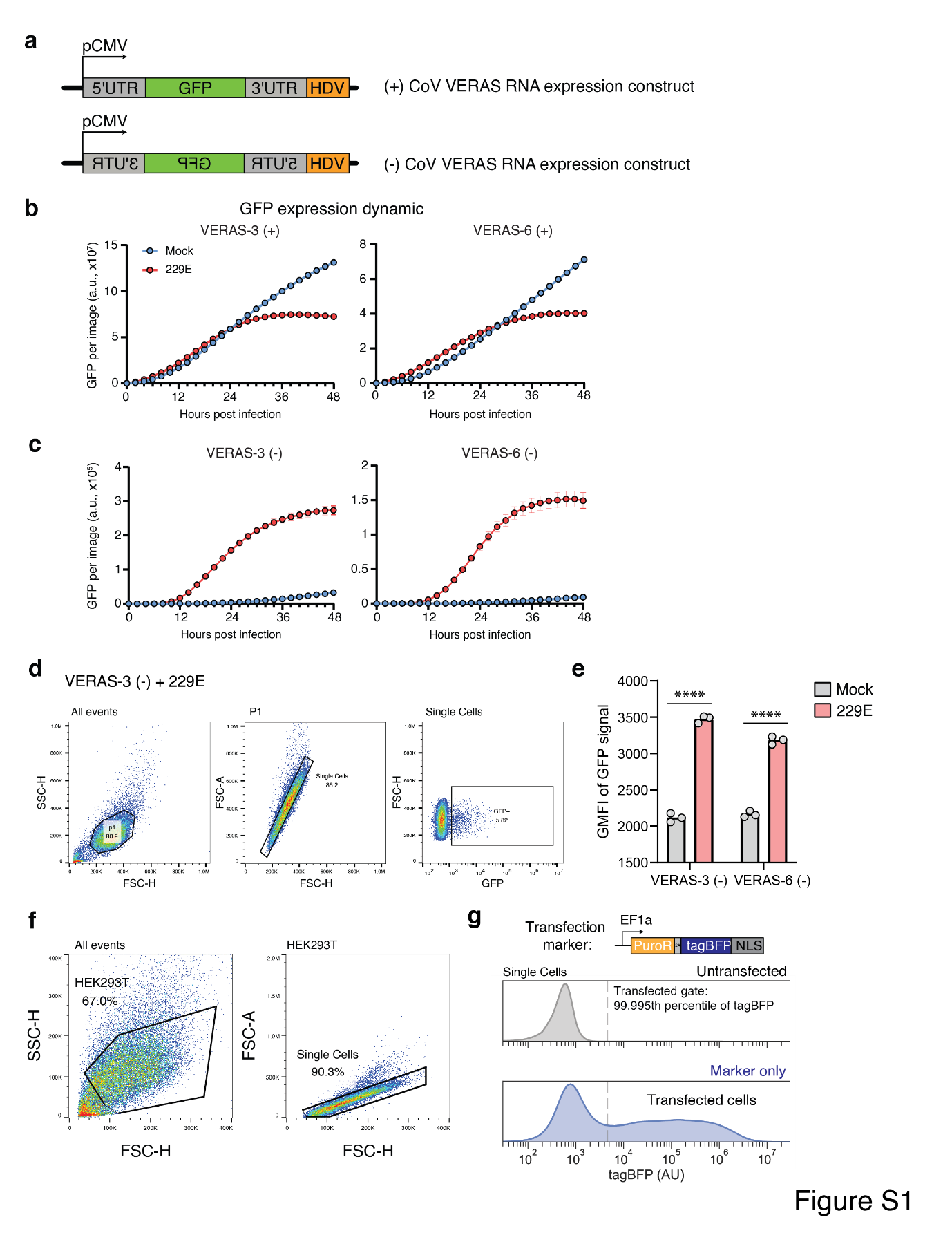


**Supplementary Figure 1 | VERASs are transcribed and replicated by human coronavirus 229E. (a)** The construct design for expression of VERAS RNAs using plasmid DNA. **(b-c)** The GFP expression dynamics after 229E infection when the cells are transfected with plasmid DNA expressing the positive-strand (**b**) or negative-strand VERASs (**c**). A total of 32 images, divided into 4 separate biological replicates, were collected at each time point and the integrated fluorescence intensity was calculated. **(d)** Gating strategy for flow cytometric analysis of GFP+ cells. (**e**) The geometric mean fluorescence intensity (GMFI) of GFP in 293T-hAPN cells, following transfection with VERAS-3 (-) and subsequent mock or 229E infection. **(f)** Gating strategy for single cells in VERAS bicistronic assay flow cytometry experiments (related to Fig. 4f). **(g)** Determination of transfected cells using tagBFP transfection marker. P values were calculated by two-tailed Student’s t-tests. ****P < 0.00001.


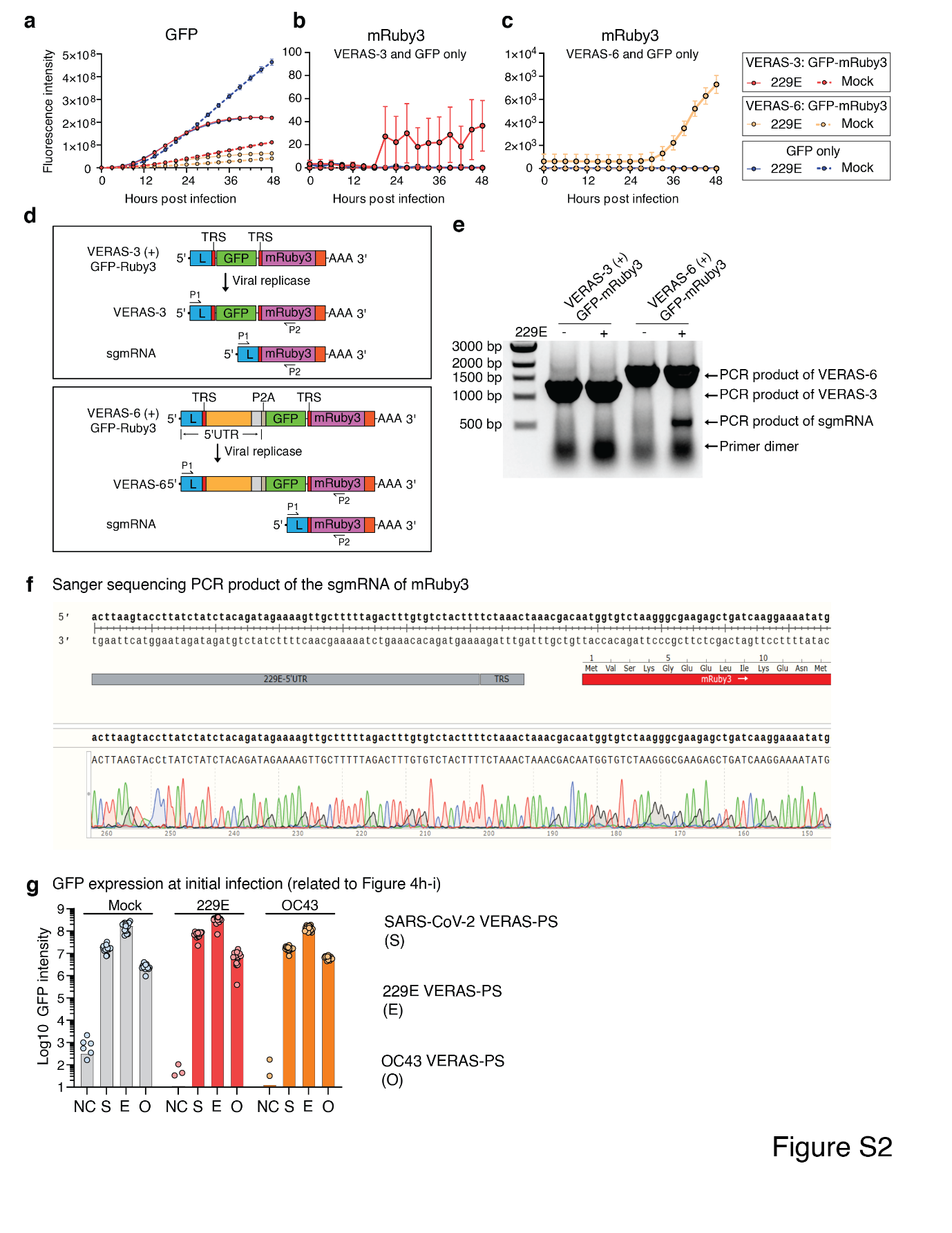


**Supplementary Figure 2 | VERASs encode a secondary protein and are packaged into progeny virions. (a-c)** The GFP and mRuby3 expression dynamics of the 293T/hAPN cells which were transfected with the VERASs and infected with or without 229E. A total of 32 images, divided into 4 separate biological replicates, were collected at each time point and the integrated GFP and RFP fluorescence intensity was calculated separately. Data are presented as mean ± s.e.m. **(d)** The method for detection of the subgenomic transcript (sgmRNA) from VERASs using RT-PCR. **(e)** The gel electrophoresis of the RT-PCR products. **(f)** The Sanger sequencing result of the RT-PCR product of the sgmRNA of mRuby3. **(g)** The GFP expression level of the cells transfected with indicated VERASs after the initial infection. 4 independent biological replicates were performed with 4 images collected per biological replicate.


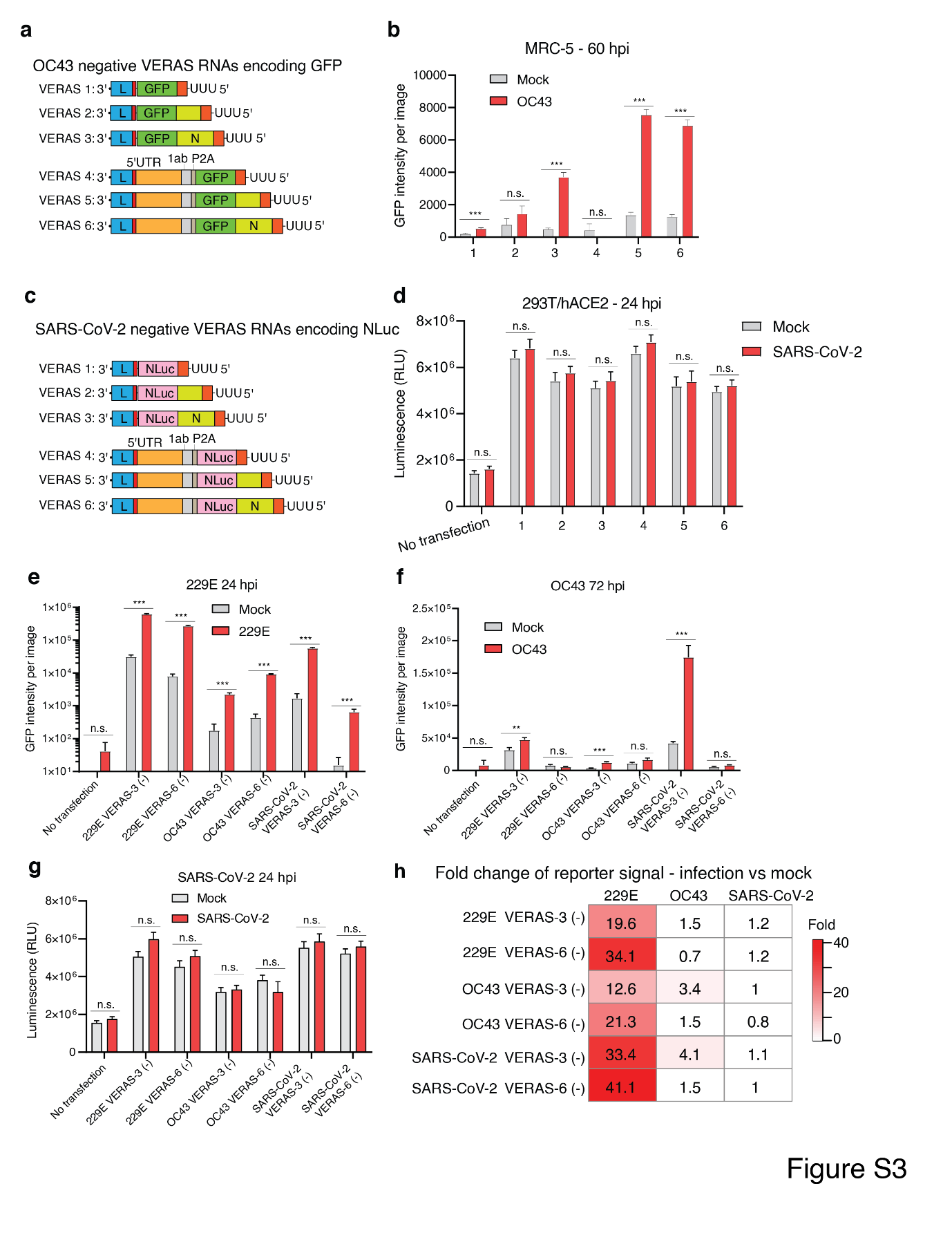


**Supplementary Figure 3 | VERASs detect and respond to broad coronaviruses.** **(a)** The VERAS designs of human coronavirus OC43 and **(b)** test of the activation of the reporter expression with OC43 infection. A total of 32 images, divided into 4 separate biological replicates, were collected and the integrated GFP fluorescence intensity was calculated. **(c)** The design of the RNA sensors of SARS-CoV-2 and **(d)** test of their effectiveness with SARS-CoV-2 infection. The luciferase activity was measured with 3 independent biological replicates. **(e-g)** The reporter activation activity of VERASs of 229E, OC43, and SARS-CoV-2 by 229E **(e),** OC43 **(f)**, and SARS-CoV-2 infection. 4 independent biological replicates were performed with 2 images collected per biological replicate (**e-f**). The luciferase activity was measured with 3 independent biological replicates (**g**). **(h)** The fold activation of the GFP signal in the infected vs mock cells expressing VERAS (-) of 229E, OC43, and SARS-CoV-2, of which the source data is plot in Fig. S3e-g. Data are presented as mean ± s.e.m. P values were calculated by two-tailed Student’s t-tests. n.s., not significant; **P < 0.01; ***P < 0.001.


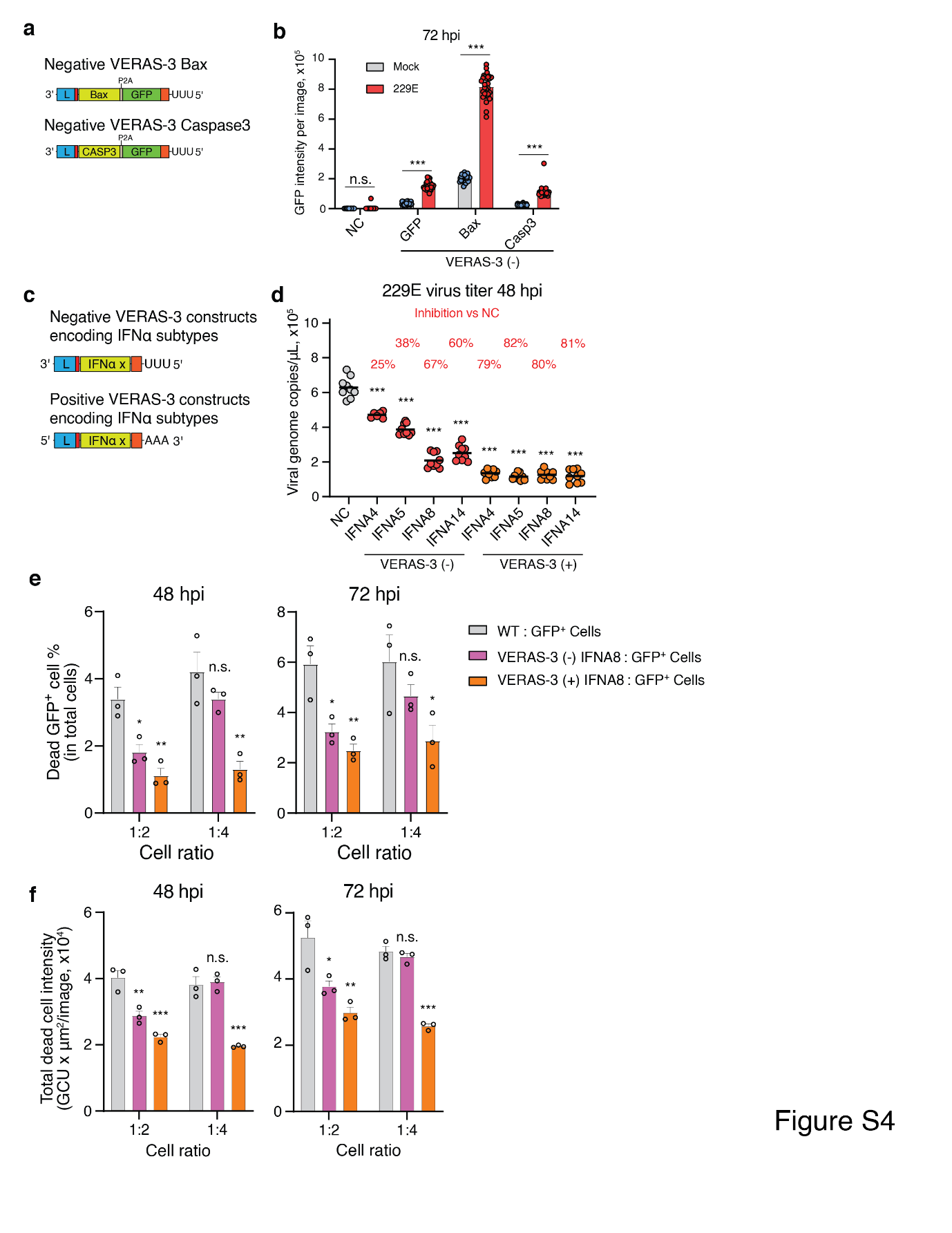


**Supplementary Figure 4 | Application of VERASs for coronavirus infection detection and antiviral treatment.** **(a)** The design of 229E VERASs encoding the apoptosis inducers, Bax and Caspase 3, and **(b)** the reporter GFP expression from these VERASs in mock or infected cells. 4 independent biological replicates were performed with 8 images per biological replicate. **(c)** The design of VERASs encoding the IFNs and **(d)** reduction of the virus titer at 48 hpi. 3 independent biological replicates were performed with 3 technical repeats per biological replicate. The bar represents the average of the group, while each circle represents an individual image. P values were calculated by two-tailed Student’s t-tests. **(e-f)** The percentage of dead GFP^+^ cells in total cells **(e)** and the total dead cell intensity **(f)** were measured at 48 and 72 hpi after the indicated mixtures of cells were challenged with 229E. 3 independent biological replicates were performed. Data are presented as mean ± s.e.m. P values were calculated by one-tailed Student’s t-tests. n.s., not significant; *P < 0.05; **P < 0.01; ***P < 0.001.

**Supplemental Information of all sequences used in this study (all sequences are annotated for components that correspond to the colors).**

**1. 229E VERAS-1 GFP (5'Leader-TRS-GFP-N-3'UTR):**

ACTTAAGTACCTTATCTATCTACAGATAGAAAAGTTGCTTTTTAGACTTTGTGTCTACTTTTCTCAACTGAACGAAAAGATGGTGAGCAAGGGCGAGGAGCTGTTCACCGGGGTGGTGCCCATCCTGGTCGAGCTGGACGGCGACGTAAACGGCCACAAGTTCAGCGTGTCCGGCGAGGGCGAGGGCGATGCCACCTACGGCAAGCTGACCCTGAAGTTCATCTGCACCACCGGCAAGCTGCCCGTGCCCTGGCCCACCCTCGTGACCACCCTGACCTACGGCGTGCAGTGCTTCAGCCGCTACCCCGACCACATGAAGCAGCACGACTTCTTCAAGTCCGCCATGCCCGAAGGCTACGTCCAGGAGCGCACCATCTTCTTCAAGGACGACGGCAACTACAAGACCCGCGCCGAGGTGAAGTTCGAGGGCGACACCCTGGTGAACCGCATCGAGCTGAAGGGCATCGACTTCAAGGAGGACGGCAACATCCTGGGGCACAAGCTGGAGTACAACTACAACAGCCACAACGTCTATATCATGGCCGACAAGCAGAAGAACGGCATCAAGGTGAACTTCAAGATCCGCCACAACATCGAGGACGGCAGCGTGCAGCTCGCCGACCACTACCAGCAGAACACCCCCATCGGCGACGGCCCCGTGCTGCTGCCCGACAACCACTACCTGAGCACCCAGTCCGCCCTGAGCAAAGACCCCAACGAGAAGCGCGATCACATGGTCCTGCTGGAGTTCGTGACCGCCGCCGGGATCACTCTCGGCATGGACGAGCTGTACAAGTAAGCTACAGTCAAATGGGCTGATGCATCTGAACCACAACGTGGTCGTCAGGGTAGAATACCTTATTCTCTTTATAGCCCTTTGCTTGTTGATAGTGAACAACCTTGGAAGGTGATACCTCGTAATTTGGTACCCATCAACAAGAAAGACAAAAATAAGCTTATAGGCTATTGGAATGTTCAAAAACGTTTCAGAACTAGAAAGGGCAAACGGGTGGATTTGTCACCCAAGCTGCATTTTTATTATCTTGGCACAGGACCCCATAAAGATGCAAAATTTAGAGAGCGTGTTGAAGGTGTCGTCTGGGTTGCTGTTGATGGTGCTAAAACTGAACCTACAGGTTACGGTGTTAGGCGCAAGAATTCAGAACCAGAGATACCACACTTCAATCAAAAGCTCCCAAATGGTGTTACTGTTGTTGAAGAACCTGACTCCCGTGCTCCTTCCCGGTCTCAGTCGAGGTCGCAGAGTCGCGGTCGTGGTGAATCCAAACCTCAATCTCGGAATCCTTCAAGTGACAGAAACCATAACAGTCAGGATGACATCATGAAGGCAGTTGCTGCGGCTCTTAAATCTTTAGGTTTTGACAAGCCTCAGGAAAAAGATAAAAAGTCAGCGAAAACGGGTACTCCTAAGCCTTCTCGTAATCAGAGTCCTGCTTCTTCTCAAACTTCTGCCAAGAGTCTTGCTCGTTCTCAGAGTTCTGAAACAAAAGAACAAAAGCATGAAATGCAAAAGCCACGGTGGAAAAGACAGCCTAATGATGATGTGACATCTAATGTCACACAATGTTTTGGCCCCAGAGACCTTGACCACAACTTTGGAAGTGCAGGTGTTGTGGCCAATGGTGTTAAAGCTAAAGGCTATCCACAATTTGCTGAGCTTGTGCCGTCAACAGCTGCTATGCTGTTTGATAGTCACATTGTTTCCAAAGAGTCAGGCAACACTGTGGTCTTGACTTTCACTACTAGAGTGACTGTGCCCAAAGACCATCCACACTTGGGTAAGTTTCTTGAGGAGTTAAATGCATTCACTAGAGAAATGCAACAACATCCTCTTCTTAACCCTAGTGCACTAGAATTCAACCCATCTCAAACTTCACCTGCAACTGCTGAACCAGTGCGTGATGAAGTTTCTATTGAAACTGACATAATTGATGAAGTAAACTAAACATGCCACTGTGTTGTTTGAAATTCAGGCTTTAGTTGGAATTTTGCTTTTGTTCTTTCTTTTATTATCTTTCTTTTGCCTGTTTTTAGAGAGATTTGGCGCCTTGGTGCCGTAGATGAATACATTGCTTTTCTCTGATCTATGTATGATGGTACGATCAGAGCTGCTTTTAATTAACATGATCCCTTGCTTTGGCTTGACAAGGATCTAGTCTTATACACAATGGTAAGCCAGTGGTAGTAAAGGTATAAGAAATTTGCTACTATGTTACTGAACCTAGGTGAACGCTAGTATAACTCATTACAAATGTGCTGGAGTAATCAAAGATCGCATTGACGAGCCAACAATGGAAGAGCCAGTCATTTGTCTTGAGACCTATCTAGTTAGTAACTGCTAATGGAACGGTTTCGATATGGATACACAAAAAAAAAAAAAAAAAAAAAAAAAAAAAA

**2. 229E VERAS-2 GFP (5'Leader-TRS-GFP-N-3'UTR):**

ACTTAAGTACCTTATCTATCTACAGATAGAAAAGTTGCTTTTTAGACTTTGTGTCTACTTTTCTCAACTGAACGAAAAGATGGTGAGCAAGGGCGAGGAGCTGTTCACCGGGGTGGTGCCCATCCTGGTCGAGCTGGACGGCGACGTAAACGGCCACAAGTTCAGCGTGTCCGGCGAGGGCGAGGGCGATGCCACCTACGGCAAGCTGACCCTGAAGTTCATCTGCACCACCGGCAAGCTGCCCGTGCCCTGGCCCACCCTCGTGACCACCCTGACCTACGGCGTGCAGTGCTTCAGCCGCTACCCCGACCACATGAAGCAGCACGACTTCTTCAAGTCCGCCATGCCCGAAGGCTACGTCCAGGAGCGCACCATCTTCTTCAAGGACGACGGCAACTACAAGACCCGCGCCGAGGTGAAGTTCGAGGGCGACACCCTGGTGAACCGCATCGAGCTGAAGGGCATCGACTTCAAGGAGGACGGCAACATCCTGGGGCACAAGCTGGAGTACAACTACAACAGCCACAACGTCTATATCATGGCCGACAAGCAGAAGAACGGCATCAAGGTGAACTTCAAGATCCGCCACAACATCGAGGACGGCAGCGTGCAGCTCGCCGACCACTACCAGCAGAACACCCCCATCGGCGACGGCCCCGTGCTGCTGCCCGACAACCACTACCTGAGCACCCAGTCCGCCCTGAGCAAAGACCCCAACGAGAAGCGCGATCACATGGTCCTGCTGGAGTTCGTGACCGCCGCCGGGATCACTCTCGGCATGGACGAGCTGTACAAGTAACCTAGTGCACTAGAATTCAACCCATCTCAAACTTCACCTGCAACTGCTGAACCAGTGCGTGATGAAGTTTCTATTGAAACTGACATAATTGATGAAGTAAACTAAACATGCCACTGTGTTGTTTGAAATTCAGGCTTTAGTTGGAATTTTGCTTTTGTTCTTTCTTTTATTATCTTTCTTTTGCCTGTTTTTAGAGAGATTTGGCGCCTTGGTGCCGTAGATGAATACATTGCTTTTCTCTGATCTATGTATGATGGTACGATCAGAGCTGCTTTTAATTAACATGATCCCTTGCTTTGGCTTGACAAGGATCTAGTCTTATACACAATGGTAAGCCAGTGGTAGTAAAGGTATAAGAAATTTGCTACTATGTTACTGAACCTAGGTGAACGCTAGTATAACTCATTACAAATGTGCTGGAGTAATCAAAGATCGCATTGACGAGCCAACAATGGAAGAGCCAGTCATTTGTCTTGAGACCTATCTAGTTAGTAACTGCTAATGGAACGGTTTCGATATGGATACACAAAAAAAAAAAAAAAAAAAAAAAAAAAAAA

**3. 229E VERAS-3 GFP (5'Leader-TRS-GFP-3'UTR):**

ACTTAAGTACCTTATCTATCTACAGATAGAAAAGTTGCTTTTTAGACTTTGTGTCTACTTTTCTCAACTGAACGAAAAGATGGTGAGCAAGGGCGAGGAGCTGTTCACCGGGGTGGTGCCCATCCTGGTCGAGCTGGACGGCGACGTAAACGGCCACAAGTTCAGCGTGTCCGGCGAGGGCGAGGGCGATGCCACCTACGGCAAGCTGACCCTGAAGTTCATCTGCACCACCGGCAAGCTGCCCGTGCCCTGGCCCACCCTCGTGACCACCCTGACCTACGGCGTGCAGTGCTTCAGCCGCTACCCCGACCACATGAAGCAGCACGACTTCTTCAAGTCCGCCATGCCCGAAGGCTACGTCCAGGAGCGCACCATCTTCTTCAAGGACGACGGCAACTACAAGACCCGCGCCGAGGTGAAGTTCGAGGGCGACACCCTGGTGAACCGCATCGAGCTGAAGGGCATCGACTTCAAGGAGGACGGCAACATCCTGGGGCACAAGCTGGAGTACAACTACAACAGCCACAACGTCTATATCATGGCCGACAAGCAGAAGAACGGCATCAAGGTGAACTTCAAGATCCGCCACAACATCGAGGACGGCAGCGTGCAGCTCGCCGACCACTACCAGCAGAACACCCCCATCGGCGACGGCCCCGTGCTGCTGCCCGACAACCACTACCTGAGCACCCAGTCCGCCCTGAGCAAAGACCCCAACGAGAAGCGCGATCACATGGTCCTGCTGGAGTTCGTGACCGCCGCCGGGATCACTCTCGGCATGGACGAGCTGTACAAGTAAACATGCCACTGTGTTGTTTGAAATTCAGGCTTTAGTTGGAATTTTGCTTTTGTTCTTTCTTTTATTATCTTTCTTTTGCCTGTTTTTAGAGAGATTTGGCGCCTTGGTGCCGTAGATGAATACATTGCTTTTCTCTGATCTATGTATGATGGTACGATCAGAGCTGCTTTTAATTAACATGATCCCTTGCTTTGGCTTGACAAGGATCTAGTCTTATACACAATGGTAAGCCAGTGGTAGTAAAGGTATAAGAAATTTGCTACTATGTTACTGAACCTAGGTGAACGCTAGTATAACTCATTACAAATGTGCTGGAGTAATCAAAGATCGCATTGACGAGCCAACAATGGAAGAGCCAGTCATTTGTCTTGAGACCTATCTAGTTAGTAACTGCTAATGGAACGGTTTCGATATGGATACACAAAAAAAAAAAAAAAAAAAAAAAAAAAAAA

**4. 229E VERAS-4 GFP (5'Leader-TRS-UTR-1ab-P2A-GFP-N-3'UTR):**

ACTTAAGTACCTTATCTATCTACAGATAGAAAAGTTGCTTTTTAGACTTTGTGTCTACTTTTCTCAACTAAACGAAATTTTTGCTATGGCCGGCATCTTTGATGCTGGAGTCGTAGTGTAATTGAAATTTCATTTGGGTTGCAACAGTTTGGAAGCAAGTGCTGTGTGTCCTAGTCTAAGGGTTTCGTGTTCCGTCACGAGATTCCATTCTACAAACGCCTTACTCGAGGTTCCGTCTCGTGTTTGTGTGGAAGCAAAGTTCTGTCTTTGTGGAAACCAGTAACTGTTCCTAATGGCCTGCAACCGTGTGACACTTGCCGTAGCAAGTGATTCTGAAATTTCTGCAAATGGCTGTTCTACTATTGCGCAAGCCGTCCGCCGTTATAGCGAGGCCGCTAGCAATGGTTTTAGGGCATGCCGATTTGTTTCATTAGATTTGCAGGATTGCATCGTTGGCATTGCAGACGATACATATGTTGGCAGCGGAGCTACTAACTTCAGCCTGCTGAAGCAGGCTGGAGACGTGGAGGAGAACCCTGGACCCGGGGTGAGCAAGGGCGAGGAGCTGTTCACCGGGGTGGTGCCCATCCTGGTCGAGCTGGACGGCGACGTAAACGGCCACAAGTTCAGCGTGTCCGGCGAGGGCGAGGGCGATGCCACCTACGGCAAGCTGACCCTGAAGTTCATCTGCACCACCGGCAAGCTGCCCGTGCCCTGGCCCACCCTCGTGACCACCCTGACCTACGGCGTGCAGTGCTTCAGCCGCTACCCCGACCACATGAAGCAGCACGACTTCTTCAAGTCCGCCATGCCCGAAGGCTACGTCCAGGAGCGCACCATCTTCTTCAAGGACGACGGCAACTACAAGACCCGCGCCGAGGTGAAGTTCGAGGGCGACACCCTGGTGAACCGCATCGAGCTGAAGGGCATCGACTTCAAGGAGGACGGCAACATCCTGGGGCACAAGCTGGAGTACAACTACAACAGCCACAACGTCTATATCATGGCCGACAAGCAGAAGAACGGCATCAAGGTGAACTTCAAGATCCGCCACAACATCGAGGACGGCAGCGTGCAGCTCGCCGACCACTACCAGCAGAACACCCCCATCGGCGACGGCCCCGTGCTGCTGCCCGACAACCACTACCTGAGCACCCAGTCCGCCCTGAGCAAAGACCCCAACGAGAAGCGCGATCACATGGTCCTGCTGGAGTTCGTGACCGCCGCCGGGATCACTCTCGGCATGGACGAGCTGTACAAGTAAGCTACAGTCAAATGGGCTGATGCATCTGAACCACAACGTGGTCGTCAGGGTAGAATACCTTATTCTCTTTATAGCCCTTTGCTTGTTGATAGTGAACAACCTTGGAAGGTGATACCTCGTAATTTGGTACCCATCAACAAGAAAGACAAAAATAAGCTTATAGGCTATTGGAATGTTCAAAAACGTTTCAGAACTAGAAAGGGCAAACGGGTGGATTTGTCACCCAAGCTGCATTTTTATTATCTTGGCACAGGACCCCATAAAGATGCAAAATTTAGAGAGCGTGTTGAAGGTGTCGTCTGGGTTGCTGTTGATGGTGCTAAAACTGAACCTACAGGTTACGGTGTTAGGCGCAAGAATTCAGAACCAGAGATACCACACTTCAATCAAAAGCTCCCAAATGGTGTTACTGTTGTTGAAGAACCTGACTCCCGTGCTCCTTCCCGGTCTCAGTCGAGGTCGCAGAGTCGCGGTCGTGGTGAATCCAAACCTCAATCTCGGAATCCTTCAAGTGACAGAAACCATAACAGTCAGGATGACATCATGAAGGCAGTTGCTGCGGCTCTTAAATCTTTAGGTTTTGACAAGCCTCAGGAAAAAGATAAAAAGTCAGCGAAAACGGGTACTCCTAAGCCTTCTCGTAATCAGAGTCCTGCTTCTTCTCAAACTTCTGCCAAGAGTCTTGCTCGTTCTCAGAGTTCTGAAACAAAAGAACAAAAGCATGAAATGCAAAAGCCACGGTGGAAAAGACAGCCTAATGATGATGTGACATCTAATGTCACACAATGTTTTGGCCCCAGAGACCTTGACCACAACTTTGGAAGTGCAGGTGTTGTGGCCAATGGTGTTAAAGCTAAAGGCTATCCACAATTTGCTGAGCTTGTGCCGTCAACAGCTGCTATGCTGTTTGATAGTCACATTGTTTCCAAAGAGTCAGGCAACACTGTGGTCTTGACTTTCACTACTAGAGTGACTGTGCCCAAAGACCATCCACACTTGGGTAAGTTTCTTGAGGAGTTAAATGCATTCACTAGAGAAATGCAACAACATCCTCTTCTTAACCCTAGTGCACTAGAATTCAACCCATCTCAAACTTCACCTGCAACTGCTGAACCAGTGCGTGATGAAGTTTCTATTGAAACTGACATAATTGATGAAGTAAACTAAACATGCCACTGTGTTGTTTGAAATTCAGGCTTTAGTTGGAATTTTGCTTTTGTTCTTTCTTTTATTATCTTTCTTTTGCCTGTTTTTAGAGAGATTTGGCGCCTTGGTGCCGTAGATGAATACATTGCTTTTCTCTGATCTATGTATGATGGTACGATCAGAGCTGCTTTTAATTAACATGATCCCTTGCTTTGGCTTGACAAGGATCTAGTCTTATACACAATGGTAAGCCAGTGGTAGTAAAGGTATAAGAAATTTGCTACTATGTTACTGAACCTAGGTGAACGCTAGTATAACTCATTACAAATGTGCTGGAGTAATCAAAGATCGCATTGACGAGCCAACAATGGAAGAGCCAGTCATTTGTCTTGAGACCTATCTAGTTAGTAACTGCTAATGGAACGGTTTCGATATGGATACACAAAAAAAAAAAAAAAAAAAAAAAAAAAAAA

**5. 229E VERAS-5 GFP (5'Leader-TRS-UTR-1ab-P2A-GFP-N-3'UTR):**

ACTTAAGTACCTTATCTATCTACAGATAGAAAAGTTGCTTTTTAGACTTTGTGTCTACTTTTCTCAACTAAACGAAATTTTTGCTATGGCCGGCATCTTTGATGCTGGAGTCGTAGTGTAATTGAAATTTCATTTGGGTTGCAACAGTTTGGAAGCAAGTGCTGTGTGTCCTAGTCTAAGGGTTTCGTGTTCCGTCACGAGATTCCATTCTACAAACGCCTTACTCGAGGTTCCGTCTCGTGTTTGTGTGGAAGCAAAGTTCTGTCTTTGTGGAAACCAGTAACTGTTCCTAATGGCCTGCAACCGTGTGACACTTGCCGTAGCAAGTGATTCTGAAATTTCTGCAAATGGCTGTTCTACTATTGCGCAAGCCGTCCGCCGTTATAGCGAGGCCGCTAGCAATGGTTTTAGGGCATGCCGATTTGTTTCATTAGATTTGCAGGATTGCATCGTTGGCATTGCAGACGATACATATGTTGGCAGCGGAGCTACTAACTTCAGCCTGCTGAAGCAGGCTGGAGACGTGGAGGAGAACCCTGGACCCGGGGTGAGCAAGGGCGAGGAGCTGTTCACCGGGGTGGTGCCCATCCTGGTCGAGCTGGACGGCGACGTAAACGGCCACAAGTTCAGCGTGTCCGGCGAGGGCGAGGGCGATGCCACCTACGGCAAGCTGACCCTGAAGTTCATCTGCACCACCGGCAAGCTGCCCGTGCCCTGGCCCACCCTCGTGACCACCCTGACCTACGGCGTGCAGTGCTTCAGCCGCTACCCCGACCACATGAAGCAGCACGACTTCTTCAAGTCCGCCATGCCCGAAGGCTACGTCCAGGAGCGCACCATCTTCTTCAAGGACGACGGCAACTACAAGACCCGCGCCGAGGTGAAGTTCGAGGGCGACACCCTGGTGAACCGCATCGAGCTGAAGGGCATCGACTTCAAGGAGGACGGCAACATCCTGGGGCACAAGCTGGAGTACAACTACAACAGCCACAACGTCTATATCATGGCCGACAAGCAGAAGAACGGCATCAAGGTGAACTTCAAGATCCGCCACAACATCGAGGACGGCAGCGTGCAGCTCGCCGACCACTACCAGCAGAACACCCCCATCGGCGACGGCCCCGTGCTGCTGCCCGACAACCACTACCTGAGCACCCAGTCCGCCCTGAGCAAAGACCCCAACGAGAAGCGCGATCACATGGTCCTGCTGGAGTTCGTGACCGCCGCCGGGATCACTCTCGGCATGGACGAGCTGTACAAGTAACCTAGTGCACTAGAATTCAACCCATCTCAAACTTCACCTGCAACTGCTGAACCAGTGCGTGATGAAGTTTCTATTGAAACTGACATAATTGATGAAGTAAACTAAACATGCCACTGTGTTGTTTGAAATTCAGGCTTTAGTTGGAATTTTGCTTTTGTTCTTTCTTTTATTATCTTTCTTTTGCCTGTTTTTAGAGAGATTTGGCGCCTTGGTGCCGTAGATGAATACATTGCTTTTCTCTGATCTATGTATGATGGTACGATCAGAGCTGCTTTTAATTAACATGATCCCTTGCTTTGGCTTGACAAGGATCTAGTCTTATACACAATGGTAAGCCAGTGGTAGTAAAGGTATAAGAAATTTGCTACTATGTTACTGAACCTAGGTGAACGCTAGTATAACTCATTACAAATGTGCTGGAGTAATCAAAGATCGCATTGACGAGCCAACAATGGAAGAGCCAGTCATTTGTCTTGAGACCTATCTAGTTAGTAACTGCTAATGGAACGGTTTCGATATGGATACACAAAAAAAAAAAAAAAAAAAAAAAAAAAAAA

**6. 229E VERAS-6 GFP (5'Leader-TRS-UTR-1ab-P2A-GFP-3'UTR):**

ACTTAAGTACCTTATCTATCTACAGATAGAAAAGTTGCTTTTTAGACTTTGTGTCTACTTTTCTCAACTAAACGAAATTTTTGCTATGGCCGGCATCTTTGATGCTGGAGTCGTAGTGTAATTGAAATTTCATTTGGGTTGCAACAGTTTGGAAGCAAGTGCTGTGTGTCCTAGTCTAAGGGTTTCGTGTTCCGTCACGAGATTCCATTCTACAAACGCCTTACTCGAGGTTCCGTCTCGTGTTTGTGTGGAAGCAAAGTTCTGTCTTTGTGGAAACCAGTAACTGTTCCTAATGGCCTGCAACCGTGTGACACTTGCCGTAGCAAGTGATTCTGAAATTTCTGCAAATGGCTGTTCTACTATTGCGCAAGCCGTCCGCCGTTATAGCGAGGCCGCTAGCAATGGTTTTAGGGCATGCCGATTTGTTTCATTAGATTTGCAGGATTGCATCGTTGGCATTGCAGACGATACATATGTTGGCAGCGGAGCTACTAACTTCAGCCTGCTGAAGCAGGCTGGAGACGTGGAGGAGAACCCTGGACCCGGGGTGAGCAAGGGCGAGGAGCTGTTCACCGGGGTGGTGCCCATCCTGGTCGAGCTGGACGGCGACGTAAACGGCCACAAGTTCAGCGTGTCCGGCGAGGGCGAGGGCGATGCCACCTACGGCAAGCTGACCCTGAAGTTCATCTGCACCACCGGCAAGCTGCCCGTGCCCTGGCCCACCCTCGTGACCACCCTGACCTACGGCGTGCAGTGCTTCAGCCGCTACCCCGACCACATGAAGCAGCACGACTTCTTCAAGTCCGCCATGCCCGAAGGCTACGTCCAGGAGCGCACCATCTTCTTCAAGGACGACGGCAACTACAAGACCCGCGCCGAGGTGAAGTTCGAGGGCGACACCCTGGTGAACCGCATCGAGCTGAAGGGCATCGACTTCAAGGAGGACGGCAACATCCTGGGGCACAAGCTGGAGTACAACTACAACAGCCACAACGTCTATATCATGGCCGACAAGCAGAAGAACGGCATCAAGGTGAACTTCAAGATCCGCCACAACATCGAGGACGGCAGCGTGCAGCTCGCCGACCACTACCAGCAGAACACCCCCATCGGCGACGGCCCCGTGCTGCTGCCCGACAACCACTACCTGAGCACCCAGTCCGCCCTGAGCAAAGACCCCAACGAGAAGCGCGATCACATGGTCCTGCTGGAGTTCGTGACCGCCGCCGGGATCACTCTCGGCATGGACGAGCTGTACAAGTAAACATGCCACTGTGTTGTTTGAAATTCAGGCTTTAGTTGGAATTTTGCTTTTGTTCTTTCTTTTATTATCTTTCTTTTGCCTGTTTTTAGAGAGATTTGGCGCCTTGGTGCCGTAGATGAATACATTGCTTTTCTCTGATCTATGTATGATGGTACGATCAGAGCTGCTTTTAATTAACATGATCCCTTGCTTTGGCTTGACAAGGATCTAGTCTTATACACAATGGTAAGCCAGTGGTAGTAAAGGTATAAGAAATTTGCTACTATGTTACTGAACCTAGGTGAACGCTAGTATAACTCATTACAAATGTGCTGGAGTAATCAAAGATCGCATTGACGAGCCAACAATGGAAGAGCCAGTCATTTGTCTTGAGACCTATCTAGTTAGTAACTGCTAATGGAACGGTTTCGATATGGATACACAAAAAAAAAAAAAAAAAAAAAAAAAAAAAA

**7. 229E VERAS-3 GFP-mRuby3 (5'Leader-TRS-GFP-TRS-mRuby3-3'UTR):**

ACTTAAGTACCTTATCTATCTACAGATAGAAAAGTTGCTTTTTAGACTTTGTGTCTACTTTTCTCAACTGAACGAAAAGATGGTGAGCAAGGGCGAGGAGCTGTTCACCGGGGTGGTGCCCATCCTGGTCGAGCTGGACGGCGACGTAAACGGCCACAAGTTCAGCGTGTCCGGCGAGGGCGAGGGCGATGCCACCTACGGCAAGCTGACCCTGAAGTTCATCTGCACCACCGGCAAGCTGCCCGTGCCCTGGCCCACCCTCGTGACCACCCTGACCTACGGCGTGCAGTGCTTCAGCCGCTACCCCGACCACATGAAGCAGCACGACTTCTTCAAGTCCGCCATGCCCGAAGGCTACGTCCAGGAGCGCACCATCTTCTTCAAGGACGACGGCAACTACAAGACCCGCGCCGAGGTGAAGTTCGAGGGCGACACCCTGGTGAACCGCATCGAGCTGAAGGGCATCGACTTCAAGGAGGACGGCAACATCCTGGGGCACAAGCTGGAGTACAACTACAACAGCCACAACGTCTATATCATGGCCGACAAGCAGAAGAACGGCATCAAGGTGAACTTCAAGATCCGCCACAACATCGAGGACGGCAGCGTGCAGCTCGCCGACCACTACCAGCAGAACACCCCCATCGGCGACGGCCCCGTGCTGCTGCCCGACAACCACTACCTGAGCACCCAGTCCGCCCTGAGCAAAGACCCCAACGAGAAGCGCGATCACATGGTCCTGCTGGAGTTCGTGACCGCCGCCGGGATCACTCTCGGCATGGACGAGCTGTACAAGTAATTCTCAACTAAACGACAATGGTGTCTAAGGGCGAAGAGCTGATCAAGGAAAATATGCGTATGAAGGTGGTCATGGAAGGTTCGGTCAACGGCCACCAATTCAAATGCACAGGTGAAGGAGAAGGCAGACCGTACGAGGGAGTGCAAACCATGAGGATCAAAGTCATCGAGGGAGGACCCCTGCCATTTGCCTTTGACATTCTTGCCACGTCGTTCATGTATGGCAGCCGTACCTTTATCAAGTACCCGGCCGACATCCCTGATTTCTTTAAACAGTCCTTTCCTGAGGGTTTTACTTGGGAAAGAGTTACGAGATACGAAGATGGTGGAGTCGTCACCGTCACGCAGGACACCAGCCTTGAGGATGGCGAGCTCGTCTACAACGTCAAGGTCAGAGGGGTAAACTTTCCCTCCAATGGTCCCGTGATGCAGAAGAAGACCAAGGGTTGGGAGCCTAATACAGAGATGATGTATCCAGCAGATGGTGGTCTGAGAGGATACACTGACATCGCACTGAAAGTTGATGGTGGTGGCCATCTGCACTGCAACTTCGTGACAACTTACAGGTCAAAAAAGACCGTCGGGAACATCAAGATGCCCGGTGTCCATGCCGTTGATCACCGCCTGGAAAGGATCGAGGAGAGTGACAATGAAACCTACGTAGTGCAAAGAGAAGTGGCAGTTGCCAAATACAGCAACCTTGGTGGTGGCATGGACGAGCTGTACAAGTAAACATGCCACTGTGTTGTTTGAAATTCAGGCTTTAGTTGGAATTTTGCTTTTGTTCTTTCTTTTATTATCTTTCTTTTGCCTGTTTTTAGAGAGATTTGGCGCCTTGGTGCCGTAGATGAATACATTGCTTTTCTCTGATCTATGTATGATGGTACGATCAGAGCTGCTTTTAATTAACATGATCCCTTGCTTTGGCTTGACAAGGATCTAGTCTTATACACAATGGTAAGCCAGTGGTAGTAAAGGTATAAGAAATTTGCTACTATGTTACTGAACCTAGGTGAACGCTAGTATAACTCATTACAAATGTGCTGGAGTAATCAAAGATCGCATTGACGAGCCAACAATGGAAGAGCCAGTCATTTGTCTTGAGACCTATCTAGTTAGTAACTGCTAATGGAACGGTTTCGATATGGATACACAAAAAAAAAAAAAAAAAAAAAAAAAAAAAA

**8. 229E VERAS-6 GFP-mRuby3 (5'Leader-TRS-UTR-1ab-P2A-GFP-TRS-mRuby3-3'UTR):**

ACTTAAGTACCTTATCTATCTACAGATAGAAAAGTTGCTTTTTAGACTTTGTGTCTACTTTTCTCAACTAAACGAAATTTTTGCTATGGCCGGCATCTTTGATGCTGGAGTCGTAGTGTAATTGAAATTTCATTTGGGTTGCAACAGTTTGGAAGCAAGTGCTGTGTGTCCTAGTCTAAGGGTTTCGTGTTCCGTCACGAGATTCCATTCTACAAACGCCTTACTCGAGGTTCCGTCTCGTGTTTGTGTGGAAGCAAAGTTCTGTCTTTGTGGAAACCAGTAACTGTTCCTAATGGCCTGCAACCGTGTGACACTTGCCGTAGCAAGTGATTCTGAAATTTCTGCAAATGGCTGTTCTACTATTGCGCAAGCCGTCCGCCGTTATAGCGAGGCCGCTAGCAATGGTTTTAGGGCATGCCGATTTGTTTCATTAGATTTGCAGGATTGCATCGTTGGCATTGCAGACGATACATATGTTGGCAGCGGAGCTACTAACTTCAGCCTGCTGAAGCAGGCTGGAGACGTGGAGGAGAACCCTGGACCCGGGGTGAGCAAGGGCGAGGAGCTGTTCACCGGGGTGGTGCCCATCCTGGTCGAGCTGGACGGCGACGTAAACGGCCACAAGTTCAGCGTGTCCGGCGAGGGCGAGGGCGATGCCACCTACGGCAAGCTGACCCTGAAGTTCATCTGCACCACCGGCAAGCTGCCCGTGCCCTGGCCCACCCTCGTGACCACCCTGACCTACGGCGTGCAGTGCTTCAGCCGCTACCCCGACCACATGAAGCAGCACGACTTCTTCAAGTCCGCCATGCCCGAAGGCTACGTCCAGGAGCGCACCATCTTCTTCAAGGACGACGGCAACTACAAGACCCGCGCCGAGGTGAAGTTCGAGGGCGACACCCTGGTGAACCGCATCGAGCTGAAGGGCATCGACTTCAAGGAGGACGGCAACATCCTGGGGCACAAGCTGGAGTACAACTACAACAGCCACAACGTCTATATCATGGCCGACAAGCAGAAGAACGGCATCAAGGTGAACTTCAAGATCCGCCACAACATCGAGGACGGCAGCGTGCAGCTCGCCGACCACTACCAGCAGAACACCCCCATCGGCGACGGCCCCGTGCTGCTGCCCGACAACCACTACCTGAGCACCCAGTCCGCCCTGAGCAAAGACCCCAACGAGAAGCGCGATCACATGGTCCTGCTGGAGTTCGTGACCGCCGCCGGGATCACTCTCGGCATGGACGAGCTGTACAAGTAATTCTCAACTAAACGACAATGGTGTCTAAGGGCGAAGAGCTGATCAAGGAAAATATGCGTATGAAGGTGGTCATGGAAGGTTCGGTCAACGGCCACCAATTCAAATGCACAGGTGAAGGAGAAGGCAGACCGTACGAGGGAGTGCAAACCATGAGGATCAAAGTCATCGAGGGAGGACCCCTGCCATTTGCCTTTGACATTCTTGCCACGTCGTTCATGTATGGCAGCCGTACCTTTATCAAGTACCCGGCCGACATCCCTGATTTCTTTAAACAGTCCTTTCCTGAGGGTTTTACTTGGGAAAGAGTTACGAGATACGAAGATGGTGGAGTCGTCACCGTCACGCAGGACACCAGCCTTGAGGATGGCGAGCTCGTCTACAACGTCAAGGTCAGAGGGGTAAACTTTCCCTCCAATGGTCCCGTGATGCAGAAGAAGACCAAGGGTTGGGAGCCTAATACAGAGATGATGTATCCAGCAGATGGTGGTCTGAGAGGATACACTGACATCGCACTGAAAGTTGATGGTGGTGGCCATCTGCACTGCAACTTCGTGACAACTTACAGGTCAAAAAAGACCGTCGGGAACATCAAGATGCCCGGTGTCCATGCCGTTGATCACCGCCTGGAAAGGATCGAGGAGAGTGACAATGAAACCTACGTAGTGCAAAGAGAAGTGGCAGTTGCCAAATACAGCAACCTTGGTGGTGGCATGGACGAGCTGTACAAGTAAACATGCCACTGTGTTGTTTGAAATTCAGGCTTTAGTTGGAATTTTGCTTTTGTTCTTTCTTTTATTATCTTTCTTTTGCCTGTTTTTAGAGAGATTTGGCGCCTTGGTGCCGTAGATGAATACATTGCTTTTCTCTGATCTATGTATGATGGTACGATCAGAGCTGCTTTTAATTAACATGATCCCTTGCTTTGGCTTGACAAGGATCTAGTCTTATACACAATGGTAAGCCAGTGGTAGTAAAGGTATAAGAAATTTGCTACTATGTTACTGAACCTAGGTGAACGCTAGTATAACTCATTACAAATGTGCTGGAGTAATCAAAGATCGCATTGACGAGCCAACAATGGAAGAGCCAGTCATTTGTCTTGAGACCTATCTAGTTAGTAACTGCTAATGGAACGGTTTCGATATGGATACACAAAAAAAAAAAAAAAAAAAAAAAAAAAAAA

**9. SARS-CoV-2 VERAS-PS (S) (5'Leader-TRS-GFP-PS-3'UTR):**

AAGGTTTATACCTTCCCAGGTAACAAACCAACCAACTTTCGATCTCTTGTAGATCTGTTCTCTAAACGAACAAACTAAAATGGTGAGCAAGGGCGAGGAGCTGTTCACCGGGGTGGTGCCCATCCTGGTCGAGCTGGACGGCGACGTAAACGGCCACAAGTTCAGCGTGTCCGGCGAGGGCGAGGGCGATGCCACCTACGGCAAGCTGACCCTGAAGTTCATCTGCACCACCGGCAAGCTGCCCGTGCCCTGGCCCACCCTCGTGACCACCCTGACCTACGGCGTGCAGTGCTTCAGCCGCTACCCCGACCACATGAAGCAGCACGACTTCTTCAAGTCCGCCATGCCCGAAGGCTACGTCCAGGAGCGCACCATCTTCTTCAAGGACGACGGCAACTACAAGACCCGCGCCGAGGTGAAGTTCGAGGGCGACACCCTGGTGAACCGCATCGAGCTGAAGGGCATCGACTTCAAGGAGGACGGCAACATCCTGGGGCACAAGCTGGAGTACAACTACAACAGCCACAACGTCTATATCATGGCCGACAAGCAGAAGAACGGCATCAAGGTGAACTTCAAGATCCGCCACAACATCGAGGACGGCAGCGTGCAGCTCGCCGACCACTACCAGCAGAACACCCCCATCGGCGACGGCCCCGTGCTGCTGCCCGACAACCACTACCTGAGCACCCAGTCCGCCCTGAGCAAAGACCCCAACGAGAAGCGCGATCACATGGTCCTGCTGGAGTTCGTGACCGCCGCCGGGATCACTCTCGGCATGGACGAGCTGTACAAGTAATAGGTCCCAAACAAGCTAGTCTTAATGGAGTCACATTAATTGGAGAAGCCGTAAAAACACAGTTCAATTATTATAAGAAAGTTGATGGTGTTGTCCAACAATTACCTGAAACTTACTTTACTCAGAGTAGAAATTTACAAGAATTTAAACCCAGGAGTCAAATGGAAATTGATTTCTTAGAATTAGCTATGGATGAATTCATTGAACGGTATAAATTAGAAGGCTATGCCTTCGAACATATCGTTTATGGAGATTTTAGTCATAGTCAGTTAGGTGGTTTACATCTACTGATTGGACTAGCTAAACGTTTTAAGGAATCACCTTTTGAATTAGAAGATTTTATTCCTATGGACAGTACAGTTAAAAACTATTTCATAACAGATGCGCAAACAGGTTCATCTAAGTGTGTGTGTTCTGTTATTGATTTATTACTTGATGATTTTGTTGAAATAATAAAATCCCAAGATTTATCTGTAGTTTCTAAGGTTGTCAAAGTGACTATTGACTATACAGAAATTTCATTTATGCTTTGGTGTAAAGATGGCCATGTAGAAACATTTTACCCAAAATTACAATCTAGTCAAGCGTGGCAACCGGGTGTTGCTATGCCTAATCTTTACAAAATGCAAAGAATGCTATTAGAAAAGTGTGACCTTCAAAATTATGGTGATAGTGCAACATTACCTAAAGGCATAATGATGAATGTCGCAAAATATACTCAACTGTGTCAATATTTAAACACATTAACATTAGCTGTACCCTATAATATGAGAGTTATACATTTTGGTGCTGGTTCTGATAAAGGAGTTGCACCAGGTACAGCTGTTTTAAGACAGTGGTTGCCTACGGGTACGCTGCTTGTCGATTCAGATCTTAATGACTTTGTCTCTGATGCAGATTCAACTTTGATTGGTGATTGTGCAACTGTACATACAGCTAATAAATGGGATCTCATTATTAGTGATATGTACGACCCTAAGACTAAAAATGTTACAAAAGAAAATGACTCTAAAGAGGGTTTTTTCACTTACATTTGTGGGTTTATACAACAAAAGCTAGCTCTTGGAGGTTCCGTGGCTATAAAGATAACAGACTCATGCAGACCACACAAGGCAGATGGGCTATATAAACGTTTTCGCTTTTCCGTTTACGATATATAGTCTACTCTTGTGCAGAATGAATTCTCGTAACTACATAGCACAAGTAGATGTAGTTAACTTTAATCTCACATAGCAATCTTTAATCAGTGTGTAACATTAGGGAGGACTTGAAAGAGCCACCACATTTTCACCGAGGCCACGCGGAGTACGATCGAGTGTACAGTGAACAATGCTAGGGAGAGCTGCCTATATGGAAGAGCCCTAATGTGTAAAATTAATTTTAGTAGTGCTATCCCCATGTGATTTTAATAGCTTCTTAGGAGAATGACGAAAAAAAAAAAAAAAAAAAAAAAAAAAAAA

**10. 229E VERAS-PS (E) (5'Leader-TRS-GFP-PS-3'UTR):**

ACTTAAGTACCTTATCTATCTACAGATAGAAAAGTTGCTTTTTAGACTTTGTGTCTACTTTTCTCAACTGAACGAAAAGATGGTGAGCAAGGGCGAGGAGCTGTTCACCGGGGTGGTGCCCATCCTGGTCGAGCTGGACGGCGACGTAAACGGCCACAAGTTCAGCGTGTCCGGCGAGGGCGAGGGCGATGCCACCTACGGCAAGCTGACCCTGAAGTTCATCTGCACCACCGGCAAGCTGCCCGTGCCCTGGCCCACCCTCGTGACCACCCTGACCTACGGCGTGCAGTGCTTCAGCCGCTACCCCGACCACATGAAGCAGCACGACTTCTTCAAGTCCGCCATGCCCGAAGGCTACGTCCAGGAGCGCACCATCTTCTTCAAGGACGACGGCAACTACAAGACCCGCGCCGAGGTGAAGTTCGAGGGCGACACCCTGGTGAACCGCATCGAGCTGAAGGGCATCGACTTCAAGGAGGACGGCAACATCCTGGGGCACAAGCTGGAGTACAACTACAACAGCCACAACGTCTATATCATGGCCGACAAGCAGAAGAACGGCATCAAGGTGAACTTCAAGATCCGCCACAACATCGAGGACGGCAGCGTGCAGCTCGCCGACCACTACCAGCAGAACACCCCCATCGGCGACGGCCCCGTGCTGCTGCCCGACAACCACTACCTGAGCACCCAGTCCGCCCTGAGCAAAGACCCCAACGAGAAGCGCGATCACATGGTCCTGCTGGAGTTCGTGACCGCCGCCGGGATCACTCTCGGCATGGACGAGCTGTACAAGTAAGTTTGATTGTTATAATTTGTGGCAGACATTCACAGAGGTCAATTTACAAGGTTTAGAGAACATTGCTTTTAACGTTGTTAATAAAGGTTCATTTGTTGGTGCTGATGGTGAATTACCAGTAGCCATTAGTGGTGATAAAGTGTTCGTACGTGATGGTAACACTGATAATTTAGTCTTTGTTAACAAAACATCACTGCCTACAAACATAGCATTTGAACTTTTTGCTAAGAGGAAGGTTGGTTTAACACCACCTCTCAGTATTCTCAAAAACCTTGGTGTTGTCGCCACATATAAGTTTGTCTTGTGGGATTATGAAGCTGAGCGTCCCTTGACAAGCTTTACTAAGTCTGTTTGTGGTTATACAGACTTTGCAGAGGATGTTTGTACTTGTTACGATAATAGTATACAAGGTTCATACGAACGTTTTACTCTGTCAACTAATGCTGTGTTATTCTCTGCTACTGCTGTGAAAACAGGTGGTAAGAGTTTGCCGGCTATTAAATTGAATTTTGGAATGCTTAATGGTAATGCAATTGCTACTGTCAAATCAGAAGATGGTAACATAAAAAATATTAACTGGTTTGTTTACGTACGCAAAGATGGCAAACCTGTTGATCATTATGATGGTTTTTATACCCAAGGTCGTAATTTACAAGACTTTTTGCCTCGCAGCACAATGGAAGAAGACTTTTTGAACATGGATATAGGCGTGTTTATTCAAAAGTATGGTCTAGAGGATTTCAACTTCGAGCACGTTGTGTATGGTGATGTTTCAAAAACTACTCTAGGCGGTTTACACTTGTTGATTTCACAAGTACGTCTGAGTAAAATGGGCATCTTAAAGGCAGAGGAGTTTGTGGCAGCATCTGACATAACACTCAAATGTTGTACTGTGACTTATCTTAATGATCCTAGTTCTAAGACTGTTTGTACTTACATGGATTTGTTGTTGGATGATTTTGTTTCTGTATTGAAGTCTTTGGATTTGACTGTTGTATCCAAGGTTCATGAGGTCATAATTGACAACAAACCATGGAGATGGATGCTATGGTGTAAAGATAATGCCGTTGCTACATTCTATCCTCAGTTGCAGAGTGCAGAATGGAAATGCGGGTATTCTATGCCTGGTATTTATAAGACACAACGTATGTGCTTAGAACCATGTAATTTGTATAATTATGGTGCAGGTTTGAAGTTGCCCAGTGGCATTATGTTCAATGTTGTTAAATACACTCAATTGTGTCAATATTTTAACAGTACCACGTTATGTGTTCCTCATAATATGAGAGTGTTACACTTGGGTGCTGGCTCTGATTATGGTGTTGCACCAGGAACTGCTGTTCTTAAAAGGTGGTTGCCGCACGACGCAATTGTTGTTGACAACGATGTTGTTGACTATGTGAGTGACGCTGATTTTAGTGTTACTGGTGATTGTGCAACCGTTTATTTGGAAGACAAGTTTGACTTGTTAATCTCTGATATGTACGATGGTAGGACAAAGGCAATTGATGGTGAAAATGTTTCGAAAGAAGGATTTTTCACTTACATCAATGGTTTCATTTGTGAAAAACTTGCCATCGGAGGTTCGATTGCTATTAAAGTAACAGAGTATAGCTGGAATAAGAAATTGTATGAACTTGTACAAAGATTTTCTTTTTGGACTATGTTTTGCACTTCTGTTAATACGTCATCATCAGAAGCCTTTGTTGTCGGAATTAACTATCTTGGTGATTTCGCACAAGGACCTTTTATAGATGGTAACATAATACACGCAAATTATGTATTTTGGCGTAACTCCACACATGCCACTGTGTTGTTTGAAATTCAGGCTTTAGTTGGAATTTTGCTTTTGTTCTTTCTTTTATTATCTTTCTTTTGCCTGTTTTTAGAGAGATTTGGCGCCTTGGTGCCGTAGATGAATACATTGCTTTTCTCTGATCTATGTATGATGGTACGATCAGAGCTGCTTTTAATTAACATGATCCCTTGCTTTGGCTTGACAAGGATCTAGTCTTATACACAATGGTAAGCCAGTGGTAGTAAAGGTATAAGAAATTTGCTACTATGTTACTGAACCTAGGTGAACGCTAGTATAACTCATTACAAATGTGCTGGAGTAATCAAAGATCGCATTGACGAGCCAACAATGGAAGAGCCAGTCATTTGTCTTGAGACCTATCTAGTTAGTAACTGCTAATGGAACGGTTTCGATATGGATACACAAAAAAAAAAAAAAAAAAAAAAAAAAAAAA

**11. OC43 VERAS-PS (O) (5'Leader-TRS-GFP-PS-N-3'UTR):**

ATTGTGAGCGATTTGCGTGCGTGCATCCCGCTTCACTGATCTCTTGTTAGATCTTTTTGTAATCTAAACTTTAAGGATGATGGTGAGCAAGGGCGAGGAGCTGTTCACCGGGGTGGTGCCCATCCTGGTCGAGCTGGACGGCGACGTAAACGGCCACAAGTTCAGCGTGTCCGGCGAGGGCGAGGGCGATGCCACCTACGGCAAGCTGACCCTGAAGTTCATCTGCACCACCGGCAAGCTGCCCGTGCCCTGGCCCACCCTCGTGACCACCCTGACCTACGGCGTGCAGTGCTTCAGCCGCTACCCCGACCACATGAAGCAGCACGACTTCTTCAAGTCCGCCATGCCCGAAGGCTACGTCCAGGAGCGCACCATCTTCTTCAAGGACGACGGCAACTACAAGACCCGCGCCGAGGTGAAGTTCGAGGGCGACACCCTGGTGAACCGCATCGAGCTGAAGGGCATCGACTTCAAGGAGGACGGCAACATCCTGGGGCACAAGCTGGAGTACAACTACAACAGCCACAACGTCTATATCATGGCCGACAAGCAGAAGAACGGCATCAAGGTGAACTTCAAGATCCGCCACAACATCGAGGACGGCAGCGTGCAGCTCGCCGACCACTACCAGCAGAACACCCCCATCGGCGACGGCCCCGTGCTGCTGCCCGACAACCACTACCTGAGCACCCAGTCCGCCCTGAGCAAAGACCCCAACGAGAAGCGCGATCACATGGTCCTGCTGGAGTTCGTGACCGCCGCCGGGATCACTCTCGGCATGGACGAGCTGTACAAGTAATTAAAGACGGTTTGTGTATGTTTTGGAACTGTAATGTGGATAAGTATCCACCGAATGCAGTTGTATGTAGATTTGACACTAGAGTGTTGAATAATTTAAATCTTCCTGGCTGTAATGGAGGTAGTTTGTATGTTAATAAACATGCATTCCACACTAAACCCTTTGCTAGGGCAGCCTTTGAGCATTTGAAGCCTATGCCATTCTTCTATTATTCAGATACGCCTTGTGTGTATATGGATGGCATGGATGCTAAGCAGGTTGATTATGTACCTTTGAAATCTGCCACGTGCATCACAAGATGCAATTTAGGTGGTGCAGTTTGTTTAAAACATGCTGAAGAGTATCGTGAGTACTTAGAGTCTTACAATACAGCTACTACAGCAGGTTTTACTTTTTGGGTCTATAAGACATTTGATTTTTATAATTTGTGGAATACGTTCACCAAGCTACAAAGCTTGGAGAATGTTGTATATAATTTAGTCAAGACTGGTCATTATACAGGACAGGCTGGTGAAATGCCTTGTGCCATTATAAATGATAAAGTTGTGGCTAAGATCGATAAGGAGGATGTTGTCATTTTTATTAATAATACAACATACCCTACTAATGTGGCCGTTGAATTATTTGCCAAGCGCAGTGTTCGACACCACCCAGAGCTTAAGCTCTTTAGAAATTTAAATATAGACGTGTGTTGGAAGCACGTCATTTGGGATTATGCTAGAGAAAGTATATTTTGCAGTAATACCTATGGTGTCTGCATGTATACAGATTTAAAGTTCATTGATAAATTGAATGTCCTTTTTGATGGTCGTGATAATGGTGCTCTTGAAGCTTTTAAACGTTCTAATAATGGCGTTTACATTTCCACGACAAAAGTTAAGAGTCTTTCGATGATAAGAGGTCCACCGCGTGCTGAATTAAATGGCGTAGTGGTGGACAAGGTTGGAGACACTGATTGTGTGTTTTATTTTGCTGTGCGTAAAGAAGGTCAGGATGTCATCTTCAGCCAATTCGACAGCCTGGGAGTCAGCTCTAACCAGAGCCCACAAGGTAATCTGGGGAGTAATGGTAAACCCGGTAATGTCGGTGGTAATGATGCTCTGTCAATCTCTACTATCTTTACACAAAGCCGTGTTATTAGCTCTTTTACATGTCGTACTGATATGGAAAAAGATTTTATAGCTTTAGATCAAGATGTGTTTATTCAGAAGTATGGTTTGGAGGACTATGCCTTTGAACACATTGTTTATGGTAACTTCAACCAGAAGATTATTGGTGGTTTGCATTTGTTAATAGGCTTGTACCGAAGACAGCAAACTTCCAATCTGGTTGTTCAGGAGTTTGTTTCATATGACTCCAGCATACACTCTTATTTTATCACTGACGAGAAGAGTGGTGGTAGTAAGAGTGTTTGCACTGTTATAGATATTTTGTTGGATGATTTTGTGGCTCTTGTTAAGTCACTTAATCTTAATTGTGTGAGTAAGGTTGTTAATGTTAATGTTGATTTTAAAGATTTTCAGTTTATGCTTTGGTGTAACGATGAGAAAGTTATGACTTTCTATCCTCGTTTGCAAGCTGCATCTGACTGGAAGCCTGGTTATTCTATGCCTGTATTATATAAGTATTTGAATTCTCCAATGGAAAGAGTTAGTCTCTGGAATTATGGGAAGCCAGTTACTTTGCCTACAGGCTGTATGATGAATGTTGCTAAGTATACTCAGTTATGTCAATATCTGAATACTACAACATTAGCTGTACCTGTTAATATGCGAGTTTTGCATTTAGGTGCAGGTTCAGAAAAAGGAGTAGCACCGGGTTCTGCAGTTCTTAGGCAGTGGTTGCCTGCTGGTACTATTCTTGTAGATAACGATTTATACCCATTTGTTAGTGACAGTGTCGCTACATATTTTGGGGATTGTATAACTTTACCCTTTGATTGTCAATGGGATTTGATAATTTCTGATATGTATGACCCTATTACTAAGAACATAGGGGCAAAAGCCGCGTGCAGCAAAATAAGAGTAGAGAGTTGACTGCAGAGGACATCAGCCTTCTTAAGAAGATGGATGAGCCCTATACTGAAGACACCTCAGAAATATAAGAGAATGAACCTTATGTCGGCATCTGGTGGTAACCCCTCGCAGAAAAGTCGAGATAAGGCACTCTCTATCAGAATGGATGTCTTGCTGCTATAATAGATAGAGAAGGTTATAGCAGACTATAGATTAATTAGTTGAAAGTTTTGTGTTGTAATGTATAGTGTTGGAGAAAGTGAAAGACTTGCGGAAGTAATTGCCGACAAGTGCCCAAGGGAAGAGCCAGCATGTTAAGTTACCACCCAGTAATTAGTAAATGAATGAAGTTAATTATGGCCAATTGGAAGAATCACAAAAAAAAAAAAAAAAAAAAAAAAA

**12. OC43 VERAS-1 GFP (5'Leader-TRS-GFP-3'UTR):**

ATTGTGAGCGATTTGCGTGCGTGCATCCCGCTTCACTGATCTCTTGTTAGATCTTTTTGTAATCTAAACTTTAAGGATGGTGAGCAAGGGCGAGGAGCTGTTCACCGGGGTGGTGCCCATCCTGGTCGAGCTGGACGGCGACGTAAACGGCCACAAGTTCAGCGTGTCCGGCGAGGGCGAGGGCGATGCCACCTACGGCAAGCTGACCCTGAAGTTCATCTGCACCACCGGCAAGCTGCCCGTGCCCTGGCCCACCCTCGTGACCACCCTGACCTACGGCGTGCAGTGCTTCAGCCGCTACCCCGACCACATGAAGCAGCACGACTTCTTCAAGTCCGCCATGCCCGAAGGCTACGTCCAGGAGCGCACCATCTTCTTCAAGGACGACGGCAACTACAAGACCCGCGCCGAGGTGAAGTTCGAGGGCGACACCCTGGTGAACCGCATCGAGCTGAAGGGCATCGACTTCAAGGAGGACGGCAACATCCTGGGGCACAAGCTGGAGTACAACTACAACAGCCACAACGTCTATATCATGGCCGACAAGCAGAAGAACGGCATCAAGGTGAACTTCAAGATCCGCCACAACATCGAGGACGGCAGCGTGCAGCTCGCCGACCACTACCAGCAGAACACCCCCATCGGCGACGGCCCCGTGCTGCTGCCCGACAACCACTACCTGAGCACCCAGTCCGCCCTGAGCAAAGACCCCAACGAGAAGCGCGATCACATGGTCCTGCTGGAGTTCGTGACCGCCGCCGGGATCACTCTCGGCATGGACGAGCTGTACAAGTAACAAAAGCCGCGTGCAGCAAAATAAGAGTAGAGAGTTGACTGCAGAGGACATCAGCCTTCTTAAGAAGATGGATGAGCCCTATACTGAAGACACCTCAGAAATATAAGAGAATGAACCTTATGTCGGCATCTGGTGGTAACCCCTCGCAGAAAAGTCGAGATAAGGCACTCTCTATCAGAATGGATGTCTTGCTGCTATAATAGATAGAGAAGGTTATAGCAGACTATAGATTAATTAGTTGAAAGTTTTGTGTTGTAATGTATAGTGTTGGAGAAAGTGAAAGACTTGCGGAAGTAATTGCCGACAAGTGCCCAAGGGAAGAGCCAGCATGTTAAGTTACCACCCAGTAATTAGTAAATGAATGAAGTTAATTATGGCCAATTGGAAGAATCACAAAAAAAAAAAAAAAAAAAAAAAAAAA

**13. OC43 VERAS-2 GFP (5'Leader-TRS-GFP-N-3'UTR):**

ATTGTGAGCGATTTGCGTGCGTGCATCCCGCTTCACTGATCTCTTGTTAGATCTTTTTGTAATCTAAACTTTAAGGATGGTGAGCAAGGGCGAGGAGCTGTTCACCGGGGTGGTGCCCATCCTGGTCGAGCTGGACGGCGACGTAAACGGCCACAAGTTCAGCGTGTCCGGCGAGGGCGAGGGCGATGCCACCTACGGCAAGCTGACCCTGAAGTTCATCTGCACCACCGGCAAGCTGCCCGTGCCCTGGCCCACCCTCGTGACCACCCTGACCTACGGCGTGCAGTGCTTCAGCCGCTACCCCGACCACATGAAGCAGCACGACTTCTTCAAGTCCGCCATGCCCGAAGGCTACGTCCAGGAGCGCACCATCTTCTTCAAGGACGACGGCAACTACAAGACCCGCGCCGAGGTGAAGTTCGAGGGCGACACCCTGGTGAACCGCATCGAGCTGAAGGGCATCGACTTCAAGGAGGACGGCAACATCCTGGGGCACAAGCTGGAGTACAACTACAACAGCCACAACGTCTATATCATGGCCGACAAGCAGAAGAACGGCATCAAGGTGAACTTCAAGATCCGCCACAACATCGAGGACGGCAGCGTGCAGCTCGCCGACCACTACCAGCAGAACACCCCCATCGGCGACGGCCCCGTGCTGCTGCCCGACAACCACTACCTGAGCACCCAGTCCGCCCTGAGCAAAGACCCCAACGAGAAGCGCGATCACATGGTCCTGCTGGAGTTCGTGACCGCCGCCGGGATCACTCTCGGCATGGACGAGCTGTACAAGTAACAGAGCCTCTAGTGCAGGATCGCGTAGTAGAGCCAATTCTGGCAATAGAACCCCTACCTCTGGTGTAACACCTGACATGGCTGATCAAATTGCTAGTCTTGTTCTGGCAAAACTTGGCAAGGATGCCACTAAACCTCAGCAAGTAACTAAGCATACTGCCAAAGAAGTCAGACAGAAAATTTTGAATAAGCCCCGCCAGAAGAGGAGCCCCAATAAACAATGCACTGTTCAGCAGTGTTTTGGTAAGAGAGGCCCTAATCAGAATTTTGGTGGTGGAGAAATGTTAAAACTTGGAACTAGTGACCCACAGTTCCCCATTCTTGCAGAACTCGCACCCACAGCTGGTGCGTTTTTCTTTGGATCAAGATTAGAGTTGGCCAAAGTGCAGAATTTATCTGGGAATCCTGACGAGCCCCAGAAGGATGTTTATGAATTGCGCTATAACGGCGCAATTAGGTTTGACAGTACACTTTCAGGTTTTGAGACCATAATGAAGGTGCTGAATGAGAATTTGAATGCCTATCAACAACAAGATGGTATGATGAATATGAGTCCAAAACCACAGCGTCAGCGTGGTCATAAGAATGGACAAGGAGAAAATGATAATATAAGTGTTGCAGTGCCCAAAAGCCGCGTGCAGCAAAATAAGAGTAGAGAGTTGACTGCAGAGGACATCAGCCTTCTTAAGAAGATGGATGAGCCCTATACTGAAGACACCTCAGAAATATAAGAGAATGAACCTTATGTCGGCATCTGGTGGTAACCCCTCGCAGAAAAGTCGAGATAAGGCACTCTCTATCAGAATGGATGTCTTGCTGCTATAATAGATAGAGAAGGTTATAGCAGACTATAGATTAATTAGTTGAAAGTTTTGTGTTGTAATGTATAGTGTTGGAGAAAGTGAAAGACTTGCGGAAGTAATTGCCGACAAGTGCCCAAGGGAAGAGCCAGCATGTTAAGTTACCACCCAGTAATTAGTAAATGAATGAAGTTAATTATGGCCAATTGGAAGAATCACAAAAAAAAAAAAAAAAAAAAAAAAAAA

**14. OC43 VERAS-3 GFP (5'Leader-TRS-GFP-N-3'UTR):**

ATTGTGAGCGATTTGCGTGCGTGCATCCCGCTTCACTGATCTCTTGTTAGATCTTTTTGTAATCTAAACTTTAAGGATGGTGAGCAAGGGCGAGGAGCTGTTCACCGGGGTGGTGCCCATCCTGGTCGAGCTGGACGGCGACGTAAACGGCCACAAGTTCAGCGTGTCCGGCGAGGGCGAGGGCGATGCCACCTACGGCAAGCTGACCCTGAAGTTCATCTGCACCACCGGCAAGCTGCCCGTGCCCTGGCCCACCCTCGTGACCACCCTGACCTACGGCGTGCAGTGCTTCAGCCGCTACCCCGACCACATGAAGCAGCACGACTTCTTCAAGTCCGCCATGCCCGAAGGCTACGTCCAGGAGCGCACCATCTTCTTCAAGGACGACGGCAACTACAAGACCCGCGCCGAGGTGAAGTTCGAGGGCGACACCCTGGTGAACCGCATCGAGCTGAAGGGCATCGACTTCAAGGAGGACGGCAACATCCTGGGGCACAAGCTGGAGTACAACTACAACAGCCACAACGTCTATATCATGGCCGACAAGCAGAAGAACGGCATCAAGGTGAACTTCAAGATCCGCCACAACATCGAGGACGGCAGCGTGCAGCTCGCCGACCACTACCAGCAGAACACCCCCATCGGCGACGGCCCCGTGCTGCTGCCCGACAACCACTACCTGAGCACCCAGTCCGCCCTGAGCAAAGACCCCAACGAGAAGCGCGATCACATGGTCCTGCTGGAGTTCGTGACCGCCGCCGGGATCACTCTCGGCATGGACGAGCTGTACAAGTAAATGTCTTTTACTCCTGGTAAGCAATCCAGTAGTAGAGCGTCCTCTGGAAATCGTTCTGGTAATGGCATCCTCAAGTGGGCCGATCAGTCCGACCAGTTTAGAAATGTTCAAACCAGGGGTAGAAGAGCTCAACCCAAGCAAACTGCTACCTCTCAGCAACCATCAGGAGGGAATGTTGTACCCTACTATTCTTGGTTCTCTGGAATTACTCAGTTTCAAAAGGGAAAGGAGTTTGAGTTTGTAGAAGGACAAGGTGTGCCTATTGCACCAGGAGTCCCAGCTACTGAAGCTAAGGGGTACTGGTACAGACACAACAGACGTTCTTTTAAAACAGCCGATGGCAACCAGCGTCAACTGCTGCCACGATGGTATTTTTACTATCTGGGAACAGGACCGCATGCTAAAGACCAGTACGGCACCGATATTGACGGAGTCTACTGGGTCGCTAGCAACCAGGCTGATGTCAATACCCCGGCTGACATTGTCGATCGGGACCCAAGTAGCGATGAGGCTATTCCGACTAGGTTTCCGCCTGGCACGGTACTCCCTCAGGGTTACTATATTGAAGGCTCAGGAAGGTCTGCTCCTAATTCCAGATCTACTTCGCGCACATCCAGCAGAGCCTCTAGTGCAGGATCGCGTAGTAGAGCCAATTCTGGCAATAGAACCCCTACCTCTGGTGTAACACCTGACATGGCTGATCAAATTGCTAGTCTTGTTCTGGCAAAACTTGGCAAGGATGCCACTAAACCTCAGCAAGTAACTAAGCATACTGCCAAAGAAGTCAGACAGAAAATTTTGAATAAGCCCCGCCAGAAGAGGAGCCCCAATAAACAATGCACTGTTCAGCAGTGTTTTGGTAAGAGAGGCCCTAATCAGAATTTTGGTGGTGGAGAAATGTTAAAACTTGGAACTAGTGACCCACAGTTCCCCATTCTTGCAGAACTCGCACCCACAGCTGGTGCGTTTTTCTTTGGATCAAGATTAGAGTTGGCCAAAGTGCAGAATTTATCTGGGAATCCTGACGAGCCCCAGAAGGATGTTTATGAATTGCGCTATAACGGCGCAATTAGGTTTGACAGTACACTTTCAGGTTTTGAGACCATAATGAAGGTGCTGAATGAGAATTTGAATGCCTATCAACAACAAGATGGTATGATGAATATGAGTCCAAAACCACAGCGTCAGCGTGGTCATAAGAATGGACAAGGAGAAAATGATAATATAAGTGTTGCAGTGCCCAAAAGCCGCGTGCAGCAAAATAAGAGTAGAGAGTTGACTGCAGAGGACATCAGCCTTCTTAAGAAGATGGATGAGCCCTATACTGAAGACACCTCAGAAATATAAGAGAATGAACCTTATGTCGGCATCTGGTGGTAACCCCTCGCAGAAAAGTCGAGATAAGGCACTCTCTATCAGAATGGATGTCTTGCTGCTATAATAGATAGAGAAGGTTATAGCAGACTATAGATTAATTAGTTGAAAGTTTTGTGTTGTAATGTATAGTGTTGGAGAAAGTGAAAGACTTGCGGAAGTAATTGCCGACAAGTGCCCAAGGGAAGAGCCAGCATGTTAAGTTACCACCCAGTAATTAGTAAATGAATGAAGTTAATTATGGCCAATTGGAAGAATCACAAAAAAAAAAAAAAAAAAAAAAAAAAA

**15. OC43 VERAS-4 GFP (5'Leader-TRS-UTR-1ab-P2A-GFP-3'UTR):**

ATTGTGAGCGATTTGCGTGCGTGCATCCCGCTTCACTGATCTCTTGTTAGATCTTTTTGTAATCTAAACTTTATAAAAACATCCACTCTCTGTAATCTATGCTTGTGGGCGTAGATTTTTCATAGTGGTGTTTATATTCATTTCTGCTGTTAACAGCTTTCAGCCAGGGACGTGTTGTATCCTAGGCAGTGGCCCGCCCATAGGTCACAATGTCGAAGATCAACAAATACGGTCTCGAACTACACTGGGCTCCAGAATTTCCATGGATGTTTGAGGACGCAGAGGAGAAGTTGGATAACCCTAGTAGTTCAGAGGTGGATATGATTTGCTCCACCACTGCGCAAAAGCTGGAAACAGACGGAATTTGTCCTGAAAATCATGTGATGGTGGATTGTCGCCGACTTCTTAAACAAGAGTGTTGTGTGCAGTCTAGCCTAATACGTGAAATTGTTATGAATGCAAGTCCATATGATGGCAGCGGAGCTACTAACTTCAGCCTGCTGAAGCAGGCTGGAGACGTGGAGGAGAACCCTGGACCCGGGATGGTGAGCAAGGGCGAGGAGCTGTTCACCGGGGTGGTGCCCATCCTGGTCGAGCTGGACGGCGACGTAAACGGCCACAAGTTCAGCGTGTCCGGCGAGGGCGAGGGCGATGCCACCTACGGCAAGCTGACCCTGAAGTTCATCTGCACCACCGGCAAGCTGCCCGTGCCCTGGCCCACCCTCGTGACCACCCTGACCTACGGCGTGCAGTGCTTCAGCCGCTACCCCGACCACATGAAGCAGCACGACTTCTTCAAGTCCGCCATGCCCGAAGGCTACGTCCAGGAGCGCACCATCTTCTTCAAGGACGACGGCAACTACAAGACCCGCGCCGAGGTGAAGTTCGAGGGCGACACCCTGGTGAACCGCATCGAGCTGAAGGGCATCGACTTCAAGGAGGACGGCAACATCCTGGGGCACAAGCTGGAGTACAACTACAACAGCCACAACGTCTATATCATGGCCGACAAGCAGAAGAACGGCATCAAGGTGAACTTCAAGATCCGCCACAACATCGAGGACGGCAGCGTGCAGCTCGCCGACCACTACCAGCAGAACACCCCCATCGGCGACGGCCCCGTGCTGCTGCCCGACAACCACTACCTGAGCACCCAGTCCGCCCTGAGCAAAGACCCCAACGAGAAGCGCGATCACATGGTCCTGCTGGAGTTCGTGACCGCCGCCGGGATCACTCTCGGCATGGACGAGCTGTACAAGTAACAAAAGCCGCGTGCAGCAAAATAAGAGTAGAGAGTTGACTGCAGAGGACATCAGCCTTCTTAAGAAGATGGATGAGCCCTATACTGAAGACACCTCAGAAATATAAGAGAATGAACCTTATGTCGGCATCTGGTGGTAACCCCTCGCAGAAAAGTCGAGATAAGGCACTCTCTATCAGAATGGATGTCTTGCTGCTATAATAGATAGAGAAGGTTATAGCAGACTATAGATTAATTAGTTGAAAGTTTTGTGTTGTAATGTATAGTGTTGGAGAAAGTGAAAGACTTGCGGAAGTAATTGCCGACAAGTGCCCAAGGGAAGAGCCAGCATGTTAAGTTACCACCCAGTAATTAGTAAATGAATGAAGTTAATTATGGCCAATTGGAAGAATCACAAAAAAAAAAAAAAAAAAAAAAAAAAA

**16. OC43 VERAS-5 GFP (5'Leader-TRS-UTR-1ab-P2A-GFP-N-3'UTR):**

ATTGTGAGCGATTTGCGTGCGTGCATCCCGCTTCACTGATCTCTTGTTAGATCTTTTTGTAATCTAAACTTTATAAAAACATCCACTCTCTGTAATCTATGCTTGTGGGCGTAGATTTTTCATAGTGGTGTTTATATTCATTTCTGCTGTTAACAGCTTTCAGCCAGGGACGTGTTGTATCCTAGGCAGTGGCCCGCCCATAGGTCACAATGTCGAAGATCAACAAATACGGTCTCGAACTACACTGGGCTCCAGAATTTCCATGGATGTTTGAGGACGCAGAGGAGAAGTTGGATAACCCTAGTAGTTCAGAGGTGGATATGATTTGCTCCACCACTGCGCAAAAGCTGGAAACAGACGGAATTTGTCCTGAAAATCATGTGATGGTGGATTGTCGCCGACTTCTTAAACAAGAGTGTTGTGTGCAGTCTAGCCTAATACGTGAAATTGTTATGAATGCAAGTCCATATGATGGCAGCGGAGCTACTAACTTCAGCCTGCTGAAGCAGGCTGGAGACGTGGAGGAGAACCCTGGACCCGGGATGGTGAGCAAGGGCGAGGAGCTGTTCACCGGGGTGGTGCCCATCCTGGTCGAGCTGGACGGCGACGTAAACGGCCACAAGTTCAGCGTGTCCGGCGAGGGCGAGGGCGATGCCACCTACGGCAAGCTGACCCTGAAGTTCATCTGCACCACCGGCAAGCTGCCCGTGCCCTGGCCCACCCTCGTGACCACCCTGACCTACGGCGTGCAGTGCTTCAGCCGCTACCCCGACCACATGAAGCAGCACGACTTCTTCAAGTCCGCCATGCCCGAAGGCTACGTCCAGGAGCGCACCATCTTCTTCAAGGACGACGGCAACTACAAGACCCGCGCCGAGGTGAAGTTCGAGGGCGACACCCTGGTGAACCGCATCGAGCTGAAGGGCATCGACTTCAAGGAGGACGGCAACATCCTGGGGCACAAGCTGGAGTACAACTACAACAGCCACAACGTCTATATCATGGCCGACAAGCAGAAGAACGGCATCAAGGTGAACTTCAAGATCCGCCACAACATCGAGGACGGCAGCGTGCAGCTCGCCGACCACTACCAGCAGAACACCCCCATCGGCGACGGCCCCGTGCTGCTGCCCGACAACCACTACCTGAGCACCCAGTCCGCCCTGAGCAAAGACCCCAACGAGAAGCGCGATCACATGGTCCTGCTGGAGTTCGTGACCGCCGCCGGGATCACTCTCGGCATGGACGAGCTGTACAAGTAACAGAGCCTCTAGTGCAGGATCGCGTAGTAGAGCCAATTCTGGCAATAGAACCCCTACCTCTGGTGTAACACCTGACATGGCTGATCAAATTGCTAGTCTTGTTCTGGCAAAACTTGGCAAGGATGCCACTAAACCTCAGCAAGTAACTAAGCATACTGCCAAAGAAGTCAGACAGAAAATTTTGAATAAGCCCCGCCAGAAGAGGAGCCCCAATAAACAATGCACTGTTCAGCAGTGTTTTGGTAAGAGAGGCCCTAATCAGAATTTTGGTGGTGGAGAAATGTTAAAACTTGGAACTAGTGACCCACAGTTCCCCATTCTTGCAGAACTCGCACCCACAGCTGGTGCGTTTTTCTTTGGATCAAGATTAGAGTTGGCCAAAGTGCAGAATTTATCTGGGAATCCTGACGAGCCCCAGAAGGATGTTTATGAATTGCGCTATAACGGCGCAATTAGGTTTGACAGTACACTTTCAGGTTTTGAGACCATAATGAAGGTGCTGAATGAGAATTTGAATGCCTATCAACAACAAGATGGTATGATGAATATGAGTCCAAAACCACAGCGTCAGCGTGGTCATAAGAATGGACAAGGAGAAAATGATAATATAAGTGTTGCAGTGCCCAAAAGCCGCGTGCAGCAAAATAAGAGTAGAGAGTTGACTGCAGAGGACATCAGCCTTCTTAAGAAGATGGATGAGCCCTATACTGAAGACACCTCAGAAATATAAGAGAATGAACCTTATGTCGGCATCTGGTGGTAACCCCTCGCAGAAAAGTCGAGATAAGGCACTCTCTATCAGAATGGATGTCTTGCTGCTATAATAGATAGAGAAGGTTATAGCAGACTATAGATTAATTAGTTGAAAGTTTTGTGTTGTAATGTATAGTGTTGGAGAAAGTGAAAGACTTGCGGAAGTAATTGCCGACAAGTGCCCAAGGGAAGAGCCAGCATGTTAAGTTACCACCCAGTAATTAGTAAATGAATGAAGTTAATTATGGCCAATTGGAAGAATCACAAAAAAAAAAAAAAAAAAAAAAAAAAA

**17. OC43 VERAS-6 GFP (5'Leader-TRS-UTR-1ab-P2A-GFP-N-3'UTR):**

ATTGTGAGCGATTTGCGTGCGTGCATCCCGCTTCACTGATCTCTTGTTAGATCTTTTTGTAATCTAAACTTTATAAAAACATCCACTCTCTGTAATCTATGCTTGTGGGCGTAGATTTTTCATAGTGGTGTTTATATTCATTTCTGCTGTTAACAGCTTTCAGCCAGGGACGTGTTGTATCCTAGGCAGTGGCCCGCCCATAGGTCACAATGTCGAAGATCAACAAATACGGTCTCGAACTACACTGGGCTCCAGAATTTCCATGGATGTTTGAGGACGCAGAGGAGAAGTTGGATAACCCTAGTAGTTCAGAGGTGGATATGATTTGCTCCACCACTGCGCAAAAGCTGGAAACAGACGGAATTTGTCCTGAAAATCATGTGATGGTGGATTGTCGCCGACTTCTTAAACAAGAGTGTTGTGTGCAGTCTAGCCTAATACGTGAAATTGTTATGAATGCAAGTCCATATGATGGCAGCGGAGCTACTAACTTCAGCCTGCTGAAGCAGGCTGGAGACGTGGAGGAGAACCCTGGACCCGGGATGGTGAGCAAGGGCGAGGAGCTGTTCACCGGGGTGGTGCCCATCCTGGTCGAGCTGGACGGCGACGTAAACGGCCACAAGTTCAGCGTGTCCGGCGAGGGCGAGGGCGATGCCACCTACGGCAAGCTGACCCTGAAGTTCATCTGCACCACCGGCAAGCTGCCCGTGCCCTGGCCCACCCTCGTGACCACCCTGACCTACGGCGTGCAGTGCTTCAGCCGCTACCCCGACCACATGAAGCAGCACGACTTCTTCAAGTCCGCCATGCCCGAAGGCTACGTCCAGGAGCGCACCATCTTCTTCAAGGACGACGGCAACTACAAGACCCGCGCCGAGGTGAAGTTCGAGGGCGACACCCTGGTGAACCGCATCGAGCTGAAGGGCATCGACTTCAAGGAGGACGGCAACATCCTGGGGCACAAGCTGGAGTACAACTACAACAGCCACAACGTCTATATCATGGCCGACAAGCAGAAGAACGGCATCAAGGTGAACTTCAAGATCCGCCACAACATCGAGGACGGCAGCGTGCAGCTCGCCGACCACTACCAGCAGAACACCCCCATCGGCGACGGCCCCGTGCTGCTGCCCGACAACCACTACCTGAGCACCCAGTCCGCCCTGAGCAAAGACCCCAACGAGAAGCGCGATCACATGGTCCTGCTGGAGTTCGTGACCGCCGCCGGGATCACTCTCGGCATGGACGAGCTGTACAAGTAAATGTCTTTTACTCCTGGTAAGCAATCCAGTAGTAGAGCGTCCTCTGGAAATCGTTCTGGTAATGGCATCCTCAAGTGGGCCGATCAGTCCGACCAGTTTAGAAATGTTCAAACCAGGGGTAGAAGAGCTCAACCCAAGCAAACTGCTACCTCTCAGCAACCATCAGGAGGGAATGTTGTACCCTACTATTCTTGGTTCTCTGGAATTACTCAGTTTCAAAAGGGAAAGGAGTTTGAGTTTGTAGAAGGACAAGGTGTGCCTATTGCACCAGGAGTCCCAGCTACTGAAGCTAAGGGGTACTGGTACAGACACAACAGACGTTCTTTTAAAACAGCCGATGGCAACCAGCGTCAACTGCTGCCACGATGGTATTTTTACTATCTGGGAACAGGACCGCATGCTAAAGACCAGTACGGCACCGATATTGACGGAGTCTACTGGGTCGCTAGCAACCAGGCTGATGTCAATACCCCGGCTGACATTGTCGATCGGGACCCAAGTAGCGATGAGGCTATTCCGACTAGGTTTCCGCCTGGCACGGTACTCCCTCAGGGTTACTATATTGAAGGCTCAGGAAGGTCTGCTCCTAATTCCAGATCTACTTCGCGCACATCCAGCAGAGCCTCTAGTGCAGGATCGCGTAGTAGAGCCAATTCTGGCAATAGAACCCCTACCTCTGGTGTAACACCTGACATGGCTGATCAAATTGCTAGTCTTGTTCTGGCAAAACTTGGCAAGGATGCCACTAAACCTCAGCAAGTAACTAAGCATACTGCCAAAGAAGTCAGACAGAAAATTTTGAATAAGCCCCGCCAGAAGAGGAGCCCCAATAAACAATGCACTGTTCAGCAGTGTTTTGGTAAGAGAGGCCCTAATCAGAATTTTGGTGGTGGAGAAATGTTAAAACTTGGAACTAGTGACCCACAGTTCCCCATTCTTGCAGAACTCGCACCCACAGCTGGTGCGTTTTTCTTTGGATCAAGATTAGAGTTGGCCAAAGTGCAGAATTTATCTGGGAATCCTGACGAGCCCCAGAAGGATGTTTATGAATTGCGCTATAACGGCGCAATTAGGTTTGACAGTACACTTTCAGGTTTTGAGACCATAATGAAGGTGCTGAATGAGAATTTGAATGCCTATCAACAACAAGATGGTATGATGAATATGAGTCCAAAACCACAGCGTCAGCGTGGTCATAAGAATGGACAAGGAGAAAATGATAATATAAGTGTTGCAGTGCCCAAAAGCCGCGTGCAGCAAAATAAGAGTAGAGAGTTGACTGCAGAGGACATCAGCCTTCTTAAGAAGATGGATGAGCCCTATACTGAAGACACCTCAGAAATATAAGAGAATGAACCTTATGTCGGCATCTGGTGGTAACCCCTCGCAGAAAAGTCGAGATAAGGCACTCTCTATCAGAATGGATGTCTTGCTGCTATAATAGATAGAGAAGGTTATAGCAGACTATAGATTAATTAGTTGAAAGTTTTGTGTTGTAATGTATAGTGTTGGAGAAAGTGAAAGACTTGCGGAAGTAATTGCCGACAAGTGCCCAAGGGAAGAGCCAGCATGTTAAGTTACCACCCAGTAATTAGTAAATGAATGAAGTTAATTATGGCCAATTGGAAGAATCACAAAAAAAAAAAAAAAAAAAAAAAAAAA

**18. SASR-CoV-2 VERAS-1 NLuc (5'Leader-TRS-NLuc-3'UTR):**

AAGGTTTATACCTTCCCAGGTAACAAACCAACCAACTTTCGATCTCTTGTAGATCTGTTCTCTAAACGAACAAACTAAAATGGTCTTCACACTCGAAGATTTCGTTGGGGACTGGCGACAGACAGCCGGCTACAACCTGGACCAAGTCCTTGAACAGGGAGGTGTGTCCAGTTTGTTTCAGAATCTCGGGGTGTCCGTAACTCCGATCCAAAGGATTGTCCTGAGCGGTGAAAATGGGCTGAAGATCGACATCCATGTCATCATCCCGTATGAAGGTCTGAGCGGCGACCAAATGGGCCAGATCGAAAAAATTTTTAAGGTGGTGTACCCTGTGGATGATCATCACTTTAAGGTGATCCTGCACTATGGCACACTGGTAATCGACGGGGTTACGCCGAACATGATCGACTATTTCGGACGGCCGTATGAAGGCATCGCCGTGTTCGACGGCAAAAAGATCACTGTAACAGGGACCCTGTGGAACGGCAACAAAATTATCGACGAGCGCCTGATCAACCCCGACGGCTCCCTGCTGTTCCGAGTAACCATCAACGGAGTGACCGGCTGGCGGCTGTGCGAACGCATTCTGGCGTAAACTCATGCAGACCACACAAGGCAGATGGGCTATATAAACGTTTTCGCTTTTCCGTTTACGATATATAGTCTACTCTTGTGCAGAATGAATTCTCGTAACTACATAGCACAAGTAGATGTAGTTAACTTTAATCTCACATAGCAATCTTTAATCAGTGTGTAACATTAGGGAGGACTTGAAAGAGCCACCACATTTTCACCGAGGCCACGCGGAGTACGATCGAGTGTACAGTGAACAATGCTAGGGAGAGCTGCCTATATGGAAGAGCCCTAATGTGTAAAATTAATTTTAGTAGTGCTATCCCCATGTGATTTTAATAGCTTCTTAGGAGAATGACGAAAAAAAAAAAAAAAAAAAA

**19. SASR-CoV-2 VERAS-2 NLuc (5'Leader-TRS-NLuc-N-3'UTR):**

AAGGTTTATACCTTCCCAGGTAACAAACCAACCAACTTTCGATCTCTTGTAGATCTGTTCTCTAAACGAACAAACTAAAATGGTCTTCACACTCGAAGATTTCGTTGGGGACTGGCGACAGACAGCCGGCTACAACCTGGACCAAGTCCTTGAACAGGGAGGTGTGTCCAGTTTGTTTCAGAATCTCGGGGTGTCCGTAACTCCGATCCAAAGGATTGTCCTGAGCGGTGAAAATGGGCTGAAGATCGACATCCATGTCATCATCCCGTATGAAGGTCTGAGCGGCGACCAAATGGGCCAGATCGAAAAAATTTTTAAGGTGGTGTACCCTGTGGATGATCATCACTTTAAGGTGATCCTGCACTATGGCACACTGGTAATCGACGGGGTTACGCCGAACATGATCGACTATTTCGGACGGCCGTATGAAGGCATCGCCGTGTTCGACGGCAAAAAGATCACTGTAACAGGGACCCTGTGGAACGGCAACAAAATTATCGACGAGCGCCTGATCAACCCCGACGGCTCCCTGCTGTTCCGAGTAACCATCAACGGAGTGACCGGCTGGCGGCTGTGCGAACGCATTCTGGCGTAATTCTCCTGCTAGAATGGCTGGCAATGGCGGTGATGCTGCTCTTGCTTTGCTGCTGCTTGACAGATTGAACCAGCTTGAGAGCAAAATGTCTGGTAAAGGCCAACAACAACAAGGCCAAACTGTCACTAAGAAATCTGCTGCTGAGGCTTCTAAGAAGCCTCGGCAAAAACGTACTGCCACTAAAGCATACAATGTAACACAAGCTTTCGGCAGACGTGGTCCAGAACAAACCCAAGGAAATTTTGGGGACCAGGAACTAATCAGACAAGGAACTGATTACAAACATTGGCCGCAAATTGCACAATTTGCCCCCAGCGCTTCAGCGTTCTTCGGAATGTCGCGCATTGGCATGGAAGTCACACCTTCGGGAACGTGGTTGACCTACACAGGTGCCATCAAATTGGATGACAAAGATCCAAATTTCAAAGATCAAGTCATTTTGCTGAATAAGCATATTGACGCATACAAAACATTCCCACCAACAGAGCCTAAAAAGGACAAAAAGAAGAAGGCTGATGAAACTCAAGCCTTACCGCAGAGACAGAAGAAACAGCAAACTGTGACTCTTCTTCCTGCTGCAGATTTGGATGATTTCTCCAAACAATTGCAACAATCCATGAGCAGTGCTGACTCAACTCAGGCCTAAACTCATGCAGACCACACAAGGCAGATGGGCTATATAAACGTTTTCGCTTTTCCGTTTACGATATATAGTCTACTCTTGTGCAGAATGAATTCTCGTAACTACATAGCACAAGTAGATGTAGTTAACTTTAATCTCACATAGCAATCTTTAATCAGTGTGTAACATTAGGGAGGACTTGAAAGAGCCACCACATTTTCACCGAGGCCACGCGGAGTACGATCGAGTGTACAGTGAACAATGCTAGGGAGAGCTGCCTATATGGAAGAGCCCTAATGTGTAAAATTAATTTTAGTAGTGCTATCCCCATGTGATTTTAATAGCTTCTTAGGAGAATGACGAAAAAAAAAAAAAAAAAAAA

**20. SASR-CoV-2 VERAS-3 NLuc (5'Leader-TRS-NLuc-N-3'UTR):**

AAGGTTTATACCTTCCCAGGTAACAAACCAACCAACTTTCGATCTCTTGTAGATCTGTTCTCTAAACGAACAAACTAAAATGGTCTTCACACTCGAAGATTTCGTTGGGGACTGGCGACAGACAGCCGGCTACAACCTGGACCAAGTCCTTGAACAGGGAGGTGTGTCCAGTTTGTTTCAGAATCTCGGGGTGTCCGTAACTCCGATCCAAAGGATTGTCCTGAGCGGTGAAAATGGGCTGAAGATCGACATCCATGTCATCATCCCGTATGAAGGTCTGAGCGGCGACCAAATGGGCCAGATCGAAAAAATTTTTAAGGTGGTGTACCCTGTGGATGATCATCACTTTAAGGTGATCCTGCACTATGGCACACTGGTAATCGACGGGGTTACGCCGAACATGATCGACTATTTCGGACGGCCGTATGAAGGCATCGCCGTGTTCGACGGCAAAAAGATCACTGTAACAGGGACCCTGTGGAACGGCAACAAAATTATCGACGAGCGCCTGATCAACCCCGACGGCTCCCTGCTGTTCCGAGTAACCATCAACGGAGTGACCGGCTGGCGGCTGTGCGAACGCATTCTGGCGTAAATGTCTGATAATGGACCCCAAAATCAGCGAAATGCACCCCGCATTACGTTTGGTGGACCCTCAGATTCAACTGGCAGTAACCAGAATGGAGAACGCAGTGGGGCGCGATCAAAACAACGTCGGCCCCAAGGTTTACCCAATAATACTGCGTCTTGGTTCACCGCTCTCACTCAACATGGCAAGGAAGACCTTAAATTCCCTCGAGGACAAGGCGTTCCAATTAACACCAATAGCAGTCCAGATGACCAAATTGGCTACTACCGAAGAGCTACCAGACGAATTCGTGGTGGTGACGGTAAAATGAAAGATCTCAGTCCAAGATGGTATTTCTACTACCTAGGAACTGGGCCAGAAGCTGGACTTCCCTATGGTGCTAACAAAGACGGCATCATATGGGTTGCAACTGAGGGAGCCTTGAATACACCAAAAGATCACATTGGCACCCGCAATCCTGCTAACAATGCTGCAATCGTGCTACAACTTCCTCAAGGAACAACATTGCCAAAAGGCTTCTACGCAGAAGGGAGCAGAGGCGGCAGTCAAGCCTCTTCTCGTTCCTCATCACGTAGTCGCAACAGTTCAAGAAATTCAACTCCAGGCAGCAGTAGGGGAACTTCTCCTGCTAGAATGGCTGGCAATGGCGGTGATGCTGCTCTTGCTTTGCTGCTGCTTGACAGATTGAACCAGCTTGAGAGCAAAATGTCTGGTAAAGGCCAACAACAACAAGGCCAAACTGTCACTAAGAAATCTGCTGCTGAGGCTTCTAAGAAGCCTCGGCAAAAACGTACTGCCACTAAAGCATACAATGTAACACAAGCTTTCGGCAGACGTGGTCCAGAACAAACCCAAGGAAATTTTGGGGACCAGGAACTAATCAGACAAGGAACTGATTACAAACATTGGCCGCAAATTGCACAATTTGCCCCCAGCGCTTCAGCGTTCTTCGGAATGTCGCGCATTGGCATGGAAGTCACACCTTCGGGAACGTGGTTGACCTACACAGGTGCCATCAAATTGGATGACAAAGATCCAAATTTCAAAGATCAAGTCATTTTGCTGAATAAGCATATTGACGCATACAAAACATTCCCACCAACAGAGCCTAAAAAGGACAAAAAGAAGAAGGCTGATGAAACTCAAGCCTTACCGCAGAGACAGAAGAAACAGCAAACTGTGACTCTTCTTCCTGCTGCAGATTTGGATGATTTCTCCAAACAATTGCAACAATCCATGAGCAGTGCTGACTCAACTCAGGCCTAAACTCATGCAGACCACACAAGGCAGATGGGCTATATAAACGTTTTCGCTTTTCCGTTTACGATATATAGTCTACTCTTGTGCAGAATGAATTCTCGTAACTACATAGCACAAGTAGATGTAGTTAACTTTAATCTCACATAGCAATCTTTAATCAGTGTGTAACATTAGGGAGGACTTGAAAGAGCCACCACATTTTCACCGAGGCCACGCGGAGTACGATCGAGTGTACAGTGAACAATGCTAGGGAGAGCTGCCTATATGGAAGAGCCCTAATGTGTAAAATTAATTTTAGTAGTGCTATCCCCATGTGATTTTAATAGCTTCTTAGGAGAATGACGAAAAAAAAAAAAAAAAAAAA

**21. SASR-CoV-2 VERAS-4 NLuc (5'Leader-TRS-UTR-1ab-P2A-NLuc-3'UTR):**

AAGGTTTATACCTTCCCAGGTAACAAACCAACCAACTTTCGATCTCTTGTAGATCTGTTCTCTAAACGAACTTTAAAATCTGTGTGGCTGTCACTCGGCTGCATGCTTAGTGCACTCACGCAGTATAATTAATAACTAATTACTGTCGTTGACAGGACACGAGTAACTCGTCTATCTTCTGCAGGCTGCTTACGGTTTCGTCCGTGTTGCAGCCGATCATCAGCACATCTAGGTTTCGTCCGGGTGTGACCGAAAGGTAAGATGGAGAGCCTTGTCCCTGGTTTCAACGAGAAAACACACGTCCAACTCAGTTTGCCTGTTTTACAGGTTCGCGACGTGCTCGTACGTGGCTTTGGAGACTCCGTGGAGGAGGTCTTATCAGAGGCACGTCAACATCTTAAAGATGGCACTTGTGGCTTAGTAGAAGTTGAAAAAGGCGTTTTGCCTCAACTTGAACAGCCCTATGTGTTCATCGGCAGCGGAGCTACTAACTTCAGCCTGCTGAAGCAGGCTGGAGACGTGGAGGAGAACCCTGGACCCGGGATGGTCTTCACACTCGAAGATTTCGTTGGGGACTGGCGACAGACAGCCGGCTACAACCTGGACCAAGTCCTTGAACAGGGAGGTGTGTCCAGTTTGTTTCAGAATCTCGGGGTGTCCGTAACTCCGATCCAAAGGATTGTCCTGAGCGGTGAAAATGGGCTGAAGATCGACATCCATGTCATCATCCCGTATGAAGGTCTGAGCGGCGACCAAATGGGCCAGATCGAAAAAATTTTTAAGGTGGTGTACCCTGTGGATGATCATCACTTTAAGGTGATCCTGCACTATGGCACACTGGTAATCGACGGGGTTACGCCGAACATGATCGACTATTTCGGACGGCCGTATGAAGGCATCGCCGTGTTCGACGGCAAAAAGATCACTGTAACAGGGACCCTGTGGAACGGCAACAAAATTATCGACGAGCGCCTGATCAACCCCGACGGCTCCCTGCTGTTCCGAGTAACCATCAACGGAGTGACCGGCTGGCGGCTGTGCGAACGCATTCTGGCGTAAACTCATGCAGACCACACAAGGCAGATGGGCTATATAAACGTTTTCGCTTTTCCGTTTACGATATATAGTCTACTCTTGTGCAGAATGAATTCTCGTAACTACATAGCACAAGTAGATGTAGTTAACTTTAATCTCACATAGCAATCTTTAATCAGTGTGTAACATTAGGGAGGACTTGAAAGAGCCACCACATTTTCACCGAGGCCACGCGGAGTACGATCGAGTGTACAGTGAACAATGCTAGGGAGAGCTGCCTATATGGAAGAGCCCTAATGTGTAAAATTAATTTTAGTAGTGCTATCCCCATGTGATTTTAATAGCTTCTTAGGAGAATGACGAAAAAAAAAAAAAAAAAAAA

**22. SASR-CoV-2 VERAS-5 NLuc (5'Leader-TRS-UTR-1ab-P2A-NLuc-N-3'UTR):**

AAGGTTTATACCTTCCCAGGTAACAAACCAACCAACTTTCGATCTCTTGTAGATCTGTTCTCTAAACGAACTTTAAAATCTGTGTGGCTGTCACTCGGCTGCATGCTTAGTGCACTCACGCAGTATAATTAATAACTAATTACTGTCGTTGACAGGACACGAGTAACTCGTCTATCTTCTGCAGGCTGCTTACGGTTTCGTCCGTGTTGCAGCCGATCATCAGCACATCTAGGTTTCGTCCGGGTGTGACCGAAAGGTAAGATGGAGAGCCTTGTCCCTGGTTTCAACGAGAAAACACACGTCCAACTCAGTTTGCCTGTTTTACAGGTTCGCGACGTGCTCGTACGTGGCTTTGGAGACTCCGTGGAGGAGGTCTTATCAGAGGCACGTCAACATCTTAAAGATGGCACTTGTGGCTTAGTAGAAGTTGAAAAAGGCGTTTTGCCTCAACTTGAACAGCCCTATGTGTTCATCGGCAGCGGAGCTACTAACTTCAGCCTGCTGAAGCAGGCTGGAGACGTGGAGGAGAACCCTGGACCCGGGATGGTCTTCACACTCGAAGATTTCGTTGGGGACTGGCGACAGACAGCCGGCTACAACCTGGACCAAGTCCTTGAACAGGGAGGTGTGTCCAGTTTGTTTCAGAATCTCGGGGTGTCCGTAACTCCGATCCAAAGGATTGTCCTGAGCGGTGAAAATGGGCTGAAGATCGACATCCATGTCATCATCCCGTATGAAGGTCTGAGCGGCGACCAAATGGGCCAGATCGAAAAAATTTTTAAGGTGGTGTACCCTGTGGATGATCATCACTTTAAGGTGATCCTGCACTATGGCACACTGGTAATCGACGGGGTTACGCCGAACATGATCGACTATTTCGGACGGCCGTATGAAGGCATCGCCGTGTTCGACGGCAAAAAGATCACTGTAACAGGGACCCTGTGGAACGGCAACAAAATTATCGACGAGCGCCTGATCAACCCCGACGGCTCCCTGCTGTTCCGAGTAACCATCAACGGAGTGACCGGCTGGCGGCTGTGCGAACGCATTCTGGCGTAATTCTCCTGCTAGAATGGCTGGCAATGGCGGTGATGCTGCTCTTGCTTTGCTGCTGCTTGACAGATTGAACCAGCTTGAGAGCAAAATGTCTGGTAAAGGCCAACAACAACAAGGCCAAACTGTCACTAAGAAATCTGCTGCTGAGGCTTCTAAGAAGCCTCGGCAAAAACGTACTGCCACTAAAGCATACAATGTAACACAAGCTTTCGGCAGACGTGGTCCAGAACAAACCCAAGGAAATTTTGGGGACCAGGAACTAATCAGACAAGGAACTGATTACAAACATTGGCCGCAAATTGCACAATTTGCCCCCAGCGCTTCAGCGTTCTTCGGAATGTCGCGCATTGGCATGGAAGTCACACCTTCGGGAACGTGGTTGACCTACACAGGTGCCATCAAATTGGATGACAAAGATCCAAATTTCAAAGATCAAGTCATTTTGCTGAATAAGCATATTGACGCATACAAAACATTCCCACCAACAGAGCCTAAAAAGGACAAAAAGAAGAAGGCTGATGAAACTCAAGCCTTACCGCAGAGACAGAAGAAACAGCAAACTGTGACTCTTCTTCCTGCTGCAGATTTGGATGATTTCTCCAAACAATTGCAACAATCCATGAGCAGTGCTGACTCAACTCAGGCCTAAACTCATGCAGACCACACAAGGCAGATGGGCTATATAAACGTTTTCGCTTTTCCGTTTACGATATATAGTCTACTCTTGTGCAGAATGAATTCTCGTAACTACATAGCACAAGTAGATGTAGTTAACTTTAATCTCACATAGCAATCTTTAATCAGTGTGTAACATTAGGGAGGACTTGAAAGAGCCACCACATTTTCACCGAGGCCACGCGGAGTACGATCGAGTGTACAGTGAACAATGCTAGGGAGAGCTGCCTATATGGAAGAGCCCTAATGTGTAAAATTAATTTTAGTAGTGCTATCCCCATGTGATTTTAATAGCTTCTTAGGAGAATGACGAAAAAAAAAAAAAAAAAAAA

**23. SASR-CoV-2 VERAS-6 NLuc (5'Leader-TRS-UTR-1ab-P2A-NLuc-N-3'UTR):**

AAGGTTTATACCTTCCCAGGTAACAAACCAACCAACTTTCGATCTCTTGTAGATCTGTTCTCTAAACGAACTTTAAAATCTGTGTGGCTGTCACTCGGCTGCATGCTTAGTGCACTCACGCAGTATAATTAATAACTAATTACTGTCGTTGACAGGACACGAGTAACTCGTCTATCTTCTGCAGGCTGCTTACGGTTTCGTCCGTGTTGCAGCCGATCATCAGCACATCTAGGTTTCGTCCGGGTGTGACCGAAAGGTAAGATGGAGAGCCTTGTCCCTGGTTTCAACGAGAAAACACACGTCCAACTCAGTTTGCCTGTTTTACAGGTTCGCGACGTGCTCGTACGTGGCTTTGGAGACTCCGTGGAGGAGGTCTTATCAGAGGCACGTCAACATCTTAAAGATGGCACTTGTGGCTTAGTAGAAGTTGAAAAAGGCGTTTTGCCTCAACTTGAACAGCCCTATGTGTTCATCGGCAGCGGAGCTACTAACTTCAGCCTGCTGAAGCAGGCTGGAGACGTGGAGGAGAACCCTGGACCCGGGATGGTCTTCACACTCGAAGATTTCGTTGGGGACTGGCGACAGACAGCCGGCTACAACCTGGACCAAGTCCTTGAACAGGGAGGTGTGTCCAGTTTGTTTCAGAATCTCGGGGTGTCCGTAACTCCGATCCAAAGGATTGTCCTGAGCGGTGAAAATGGGCTGAAGATCGACATCCATGTCATCATCCCGTATGAAGGTCTGAGCGGCGACCAAATGGGCCAGATCGAAAAAATTTTTAAGGTGGTGTACCCTGTGGATGATCATCACTTTAAGGTGATCCTGCACTATGGCACACTGGTAATCGACGGGGTTACGCCGAACATGATCGACTATTTCGGACGGCCGTATGAAGGCATCGCCGTGTTCGACGGCAAAAAGATCACTGTAACAGGGACCCTGTGGAACGGCAACAAAATTATCGACGAGCGCCTGATCAACCCCGACGGCTCCCTGCTGTTCCGAGTAACCATCAACGGAGTGACCGGCTGGCGGCTGTGCGAACGCATTCTGGCGTAAATGTCTGATAATGGACCCCAAAATCAGCGAAATGCACCCCGCATTACGTTTGGTGGACCCTCAGATTCAACTGGCAGTAACCAGAATGGAGAACGCAGTGGGGCGCGATCAAAACAACGTCGGCCCCAAGGTTTACCCAATAATACTGCGTCTTGGTTCACCGCTCTCACTCAACATGGCAAGGAAGACCTTAAATTCCCTCGAGGACAAGGCGTTCCAATTAACACCAATAGCAGTCCAGATGACCAAATTGGCTACTACCGAAGAGCTACCAGACGAATTCGTGGTGGTGACGGTAAAATGAAAGATCTCAGTCCAAGATGGTATTTCTACTACCTAGGAACTGGGCCAGAAGCTGGACTTCCCTATGGTGCTAACAAAGACGGCATCATATGGGTTGCAACTGAGGGAGCCTTGAATACACCAAAAGATCACATTGGCACCCGCAATCCTGCTAACAATGCTGCAATCGTGCTACAACTTCCTCAAGGAACAACATTGCCAAAAGGCTTCTACGCAGAAGGGAGCAGAGGCGGCAGTCAAGCCTCTTCTCGTTCCTCATCACGTAGTCGCAACAGTTCAAGAAATTCAACTCCAGGCAGCAGTAGGGGAACTTCTCCTGCTAGAATGGCTGGCAATGGCGGTGATGCTGCTCTTGCTTTGCTGCTGCTTGACAGATTGAACCAGCTTGAGAGCAAAATGTCTGGTAAAGGCCAACAACAACAAGGCCAAACTGTCACTAAGAAATCTGCTGCTGAGGCTTCTAAGAAGCCTCGGCAAAAACGTACTGCCACTAAAGCATACAATGTAACACAAGCTTTCGGCAGACGTGGTCCAGAACAAACCCAAGGAAATTTTGGGGACCAGGAACTAATCAGACAAGGAACTGATTACAAACATTGGCCGCAAATTGCACAATTTGCCCCCAGCGCTTCAGCGTTCTTCGGAATGTCGCGCATTGGCATGGAAGTCACACCTTCGGGAACGTGGTTGACCTACACAGGTGCCATCAAATTGGATGACAAAGATCCAAATTTCAAAGATCAAGTCATTTTGCTGAATAAGCATATTGACGCATACAAAACATTCCCACCAACAGAGCCTAAAAAGGACAAAAAGAAGAAGGCTGATGAAACTCAAGCCTTACCGCAGAGACAGAAGAAACAGCAAACTGTGACTCTTCTTCCTGCTGCAGATTTGGATGATTTCTCCAAACAATTGCAACAATCCATGAGCAGTGCTGACTCAACTCAGGCCTAAACTCATGCAGACCACACAAGGCAGATGGGCTATATAAACGTTTTCGCTTTTCCGTTTACGATATATAGTCTACTCTTGTGCAGAATGAATTCTCGTAACTACATAGCACAAGTAGATGTAGTTAACTTTAATCTCACATAGCAATCTTTAATCAGTGTGTAACATTAGGGAGGACTTGAAAGAGCCACCACATTTTCACCGAGGCCACGCGGAGTACGATCGAGTGTACAGTGAACAATGCTAGGGAGAGCTGCCTATATGGAAGAGCCCTAATGTGTAAAATTAATTTTAGTAGTGCTATCCCCATGTGATTTTAATAGCTTCTTAGGAGAATGACGAAAAAAAAAAAAAAAAAAAA

**24. 229E VERAS-3 Bax-P2A-GFP (5'Leader-TRS-Bax-P2A-GFP-3'UTR):**

ACTTAAGTACCTTATCTATCTACAGATAGAAAAGTTGCTTTTTAGACTTTGTGTCTACTTTTCTCAACTGAACGAAAAGATGGACGGGTCCGGGGAGCAGCCCAGAGGCGGGGGGCCCACCAGCTCTGAGCAGATCATGAAGACAGGGGCCCTTTTGCTTCAGGGTTTCATCCAGGATCGAGCAGGGCGAATGGGGGGGGAGGCACCCGAGCTGGCCCTGGACCCGGTGCCTCAGGATGCGTCCACCAAGAAGCTGAGCGAGTGTCTCAAGCGCATCGGGGACGAACTGGACAGTAACATGGAGCTGCAGAGGATGATTGCCGCCGTGGACACAGACTCCCCCCGAGAGGTCTTTTTCCGAGTGGCAGCTGACATGTTTTCTGACGGCAACTTCAACTGGGGCCGGGTTGTCGCCCTTTTCTACTTTGCCAGCAAACTGGTGCTCAAGGCCCTGTGCACCAAGGTGCCGGAACTGATCAGAACCATCATGGGCTGGACATTGGACTTCCTCCGGGAGCGGCTGTTGGGCTGGATCCAAGACCAGGGTGGTTGGGACGGCCTCCTCTCCTACTTTGGGACGCCCACGTGGCAGACCGTGACCATCTTTGTGGCGGGAGTGCTCACCGCCTCACTCACCATCTGGAAGAAGATGGGCGGCAGCGGAGCTACTAACTTCAGCCTGCTGAAGCAGGCTGGAGACGTGGAGGAGAACCCTGGACCCGGGGTGAGCAAGGGCGAGGAGCTGTTCACCGGGGTGGTGCCCATCCTGGTCGAGCTGGACGGCGACGTAAACGGCCACAAGTTCAGCGTGTCCGGCGAGGGCGAGGGCGATGCCACCTACGGCAAGCTGACCCTGAAGTTCATCTGCACCACCGGCAAGCTGCCCGTGCCCTGGCCCACCCTCGTGACCACCCTGACCTACGGCGTGCAGTGCTTCAGCCGCTACCCCGACCACATGAAGCAGCACGACTTCTTCAAGTCCGCCATGCCCGAAGGCTACGTCCAGGAGCGCACCATCTTCTTCAAGGACGACGGCAACTACAAGACCCGCGCCGAGGTGAAGTTCGAGGGCGACACCCTGGTGAACCGCATCGAGCTGAAGGGCATCGACTTCAAGGAGGACGGCAACATCCTGGGGCACAAGCTGGAGTACAACTACAACAGCCACAACGTCTATATCATGGCCGACAAGCAGAAGAACGGCATCAAGGTGAACTTCAAGATCCGCCACAACATCGAGGACGGCAGCGTGCAGCTCGCCGACCACTACCAGCAGAACACCCCCATCGGCGACGGCCCCGTGCTGCTGCCCGACAACCACTACCTGAGCACCCAGTCCGCCCTGAGCAAAGACCCCAACGAGAAGCGCGATCACATGGTCCTGCTGGAGTTCGTGACCGCCGCCGGGATCACTCTCGGCATGGACGAGCTGTACAAGTAAACATGCCACTGTGTTGTTTGAAATTCAGGCTTTAGTTGGAATTTTGCTTTTGTTCTTTCTTTTATTATCTTTCTTTTGCCTGTTTTTAGAGAGATTTGGCGCCTTGGTGCCGTAGATGAATACATTGCTTTTCTCTGATCTATGTATGATGGTACGATCAGAGCTGCTTTTAATTAACATGATCCCTTGCTTTGGCTTGACAAGGATCTAGTCTTATACACAATGGTAAGCCAGTGGTAGTAAAGGTATAAGAAATTTGCTACTATGTTACTGAACCTAGGTGAACGCTAGTATAACTCATTACAAATGTGCTGGAGTAATCAAAGATCGCATTGACGAGCCAACAATGGAAGAGCCAGTCATTTGTCTTGAGACCTATCTAGTTAGTAACTGCTAATGGAACGGTTTCGATATGGATACACAAAAAAAAAAAAAAAAAAAAAAAAAAAAAA

**25. 229E VERAS-3 Caspase3(V266E)-P2A-GFP (5'Leader-TRS-Casp3-P2A-GFP-3'UTR):**

ACTTAAGTACCTTATCTATCTACAGATAGAAAAGTTGCTTTTTAGACTTTGTGTCTACTTTTCTCAACTGAACGAAAAGATGGAGAACACTGAAAACTCAGTGGATTCAAAATCCATTAAAAATTTGGAACCAAAGATCATACATGGAAGCGAATCAATGGACTCTGGAATATCCCTGGACAACAGTTATAAAATGGATTATCCTGAGATGGGTTTATGTATAATAATTAATAATAAGAATTTTCATAAAAGCACTGGAATGACATCTCGGTCTGGTACAGATGTCGATGCAGCAAACCTCAGGGAAACATTCAGAAACTTGAAATATGAAGTCAGGAATAAAAATGATCTTACACGTGAAGAAATTGTGGAATTGATGCGTGATGTTTCTAAAGAAGATCACAGCAAAAGGAGCAGTTTTGTTTGTGTGCTTCTGAGCCATGGTGAAGAAGGAATAATTTTTGGAACAAATGGACCTGTTGACCTGAAAAAAATAACAAACTTTTTCAGAGGGGATCGTTGTAGAAGTCTAACTGGAAAACCCAAACTTTTCATTATTCAGGCCTGCCGTGGTACAGAACTGGACTGTGGCATTGAGACAGACAGTGGTGTTGATGATGACATGGCGTGTCATAAAATACCAGTGGAGGCCGACTTCTTGTATGCATACTCCACAGCACCTGGTTATTATTCTTGGCGAAATTCAAAGGATGGCTCCTGGTTCATCCAGTCGCTTTGTGCCATGCTGAAACAGTATGCCGACAAGCTTGAATTTATGCACATTCTTACCCGGGTTAACCGAAAGGTGGCAACAGAATTTGAGTCCTTTTCCTTTGACGCTACTTTTCATGCAAAGAAACAGATTCCATGTATTGAGTCCATGCTCACAAAAGAACTCTATTTTTATCACGGCAGCGGAGCTACTAACTTCAGCCTGCTGAAGCAGGCTGGAGACGTGGAGGAGAACCCTGGACCCGGGGTGAGCAAGGGCGAGGAGCTGTTCACCGGGGTGGTGCCCATCCTGGTCGAGCTGGACGGCGACGTAAACGGCCACAAGTTCAGCGTGTCCGGCGAGGGCGAGGGCGATGCCACCTACGGCAAGCTGACCCTGAAGTTCATCTGCACCACCGGCAAGCTGCCCGTGCCCTGGCCCACCCTCGTGACCACCCTGACCTACGGCGTGCAGTGCTTCAGCCGCTACCCCGACCACATGAAGCAGCACGACTTCTTCAAGTCCGCCATGCCCGAAGGCTACGTCCAGGAGCGCACCATCTTCTTCAAGGACGACGGCAACTACAAGACCCGCGCCGAGGTGAAGTTCGAGGGCGACACCCTGGTGAACCGCATCGAGCTGAAGGGCATCGACTTCAAGGAGGACGGCAACATCCTGGGGCACAAGCTGGAGTACAACTACAACAGCCACAACGTCTATATCATGGCCGACAAGCAGAAGAACGGCATCAAGGTGAACTTCAAGATCCGCCACAACATCGAGGACGGCAGCGTGCAGCTCGCCGACCACTACCAGCAGAACACCCCCATCGGCGACGGCCCCGTGCTGCTGCCCGACAACCACTACCTGAGCACCCAGTCCGCCCTGAGCAAAGACCCCAACGAGAAGCGCGATCACATGGTCCTGCTGGAGTTCGTGACCGCCGCCGGGATCACTCTCGGCATGGACGAGCTGTACAAGTAAACATGCCACTGTGTTGTTTGAAATTCAGGCTTTAGTTGGAATTTTGCTTTTGTTCTTTCTTTTATTATCTTTCTTTTGCCTGTTTTTAGAGAGATTTGGCGCCTTGGTGCCGTAGATGAATACATTGCTTTTCTCTGATCTATGTATGATGGTACGATCAGAGCTGCTTTTAATTAACATGATCCCTTGCTTTGGCTTGACAAGGATCTAGTCTTATACACAATGGTAAGCCAGTGGTAGTAAAGGTATAAGAAATTTGCTACTATGTTACTGAACCTAGGTGAACGCTAGTATAACTCATTACAAATGTGCTGGAGTAATCAAAGATCGCATTGACGAGCCAACAATGGAAGAGCCAGTCATTTGTCTTGAGACCTATCTAGTTAGTAACTGCTAATGGAACGGTTTCGATATGGATACACAAAAAAAAAAAAAAAAAAAAAAAAAAAAAA

**26. 229E VERAS-3 IFNA4 (5'Leader-TRS-IFN4-3'UTR):**

ACTTAAGTACCTTATCTATCTACAGATAGAAAAGTTGCTTTTTAGACTTTGTGTCTACTTTTCTCAACTGAACGAAAAGATGGCCCTGTCCTTTTCTTTACTGATGGCCGTGCTGGTGCTCAGCTACAAATCCATCTGTTCTCTGGGCTGTGATCTGCCTCAGACCCACAGCCTGGGTAATAGGAGGGCCTTGATACTCCTGGCACAAATGGGAAGAATCTCTCATTTCTCCTGCCTGAAGGACAGACATGATTTCGGATTCCCCGAGGAGGAGTTTGATGGCCACCAGTTCCAGAAGGCTCAAGCCATCTCTGTCCTCCATGAGATGATCCAGCAGACCTTCAATCTCTTCAGCACAGAGGACTCATCTGCTGCTTGGGAACAGAGCCTCCTAGAAAAATTTTCCACTGAACTTTACCAGCAACTGAATGACCTGGAAGCATGTGTGATACAGGAGGTTGGGGTGGAAGAGACTCCCCTGATGAATGAGGACTCCATCCTGGCTGTGAGGAAATACTTCCAAAGAATCACTCTTTATCTAACAGAGAAGAAATACAGCCCTTGTGCCTGGGAGGTTGTCAGAGCAGAAATCATGAGATCCCTCTCGTTTTCAACAAACTTGCAAAAAAGATTAAGGAGGAAGGATTGAACATGCCACTGTGTTGTTTGAAATTCAGGCTTTAGTTGGAATTTTGCTTTTGTTCTTTCTTTTATTATCTTTCTTTTGCCTGTTTTTAGAGAGATTTGGCGCCTTGGTGCCGTAGATGAATACATTGCTTTTCTCTGATCTATGTATGATGGTACGATCAGAGCTGCTTTTAATTAACATGATCCCTTGCTTTGGCTTGACAAGGATCTAGTCTTATACACAATGGTAAGCCAGTGGTAGTAAAGGTATAAGAAATTTGCTACTATGTTACTGAACCTAGGTGAACGCTAGTATAACTCATTACAAATGTGCTGGAGTAATCAAAGATCGCATTGACGAGCCAACAATGGAAGAGCCAGTCATTTGTCTTGAGACCTATCTAGTTAGTAACTGCTAATGGAACGGTTTCGATATGGATACACAAAAAAAAAAAAAAAAAAAAAAAAAAAAAA

**27. 229E VERAS-3 IFNA5 (5'Leader-TRS-IFN5-3'UTR):**

ACTTAAGTACCTTATCTATCTACAGATAGAAAAGTTGCTTTTTAGACTTTGTGTCTACTTTTCTCAACTGAACGAAAAGATGGCCTTGCCCTTTGTTTTACTGATGGCCCTGGTGGTGCTCAACTGCAAGTCAATCTGTTCTCTGGGCTGTGATCTGCCTCAGACCCACAGCCTGAGTAACAGGAGGACTTTGATGATAATGGCACAAATGGGAAGAATCTCTCCTTTCTCCTGCCTGAAGGACAGACATGACTTTGGATTTCCTCAGGAGGAGTTTGATGGCAACCAGTTCCAGAAGGCTCAAGCCATCTCTGTCCTCCATGAGATGATCCAGCAGACCTTCAATCTCTTCAGCACAAAGGACTCATCTGCTACTTGGGATGAGACACTTCTAGACAAATTCTACACTGAACTTTACCAGCAGCTGAATGACCTGGAAGCCTGTATGATGCAGGAGGTTGGAGTGGAAGACACTCCTCTGATGAATGTGGACTCTATCCTGACTGTGAGAAAATACTTTCAAAGAATCACCCTCTATCTGACAGAGAAGAAATACAGCCCTTGTGCATGGGAGGTTGTCAGAGCAGAAATCATGAGATCCTTCTCTTTATCAGCAAACTTGCAAGAAAGATTAAGGAGGAAGGAATGAACATGCCACTGTGTTGTTTGAAATTCAGGCTTTAGTTGGAATTTTGCTTTTGTTCTTTCTTTTATTATCTTTCTTTTGCCTGTTTTTAGAGAGATTTGGCGCCTTGGTGCCGTAGATGAATACATTGCTTTTCTCTGATCTATGTATGATGGTACGATCAGAGCTGCTTTTAATTAACATGATCCCTTGCTTTGGCTTGACAAGGATCTAGTCTTATACACAATGGTAAGCCAGTGGTAGTAAAGGTATAAGAAATTTGCTACTATGTTACTGAACCTAGGTGAACGCTAGTATAACTCATTACAAATGTGCTGGAGTAATCAAAGATCGCATTGACGAGCCAACAATGGAAGAGCCAGTCATTTGTCTTGAGACCTATCTAGTTAGTAACTGCTAATGGAACGGTTTCGATATGGATACACAAAAAAAAAAAAAAAAAAAAAAAAAAAAAA

**28. 229E VERAS-3 IFNA8 (5'Leader-TRS-IFN8-3'UTR):**

ACTTAAGTACCTTATCTATCTACAGATAGAAAAGTTGCTTTTTAGACTTTGTGTCTACTTTTCTCAACTGAACGAAAAGATGGCCTTGACTTTTTATTTACTGGTGGCCCTAGTGGTGCTCAGCTACAAGTCATTCAGCTCTCTGGGCTGTGATCTGCCTCAGACTCACAGCCTGGGTAACAGGAGGGCCTTGATACTCCTGGCACAAATGCGAAGAATCTCTCCTTTCTCCTGCCTGAAGGACAGACATGACTTTGAATTCCCCCAGGAGGAGTTTGATGATAAACAGTTCCAGAAGGCTCAAGCCATCTCTGTCCTCCATGAGATGATCCAGCAGACCTTCAACCTCTTCAGCACAAAGGACTCATCTGCTGCTTTGGATGAGACCCTTCTAGATGAATTCTACATCGAACTTGACCAGCAGCTGAATGACCTGGAGTCCTGTGTGATGCAGGAAGTGGGGGTGATAGAGTCTCCCCTGATGTACGAGGACTCCATCCTGGCTGTGAGGAAATACTTCCAAAGAATCACTCTATATCTGACAGAGAAGAAATACAGCTCTTGTGCCTGGGAGGTTGTCAGAGCAGAAATCATGAGATCCTTCTCTTTATCAATCAACTTGCAAAAAAGATTGAAGAGTAAGGAAGGATCCGATTATAAGGATCACGATGGAGATTACAAGGACCACGACATAGACTACAAAGATGATGACGACAAGTAAACATGCCACTGTGTTGTTTGAAATTCAGGCTTTAGTTGGAATTTTGCTTTTGTTCTTTCTTTTATTATCTTTCTTTTGCCTGTTTTTAGAGAGATTTGGCGCCTTGGTGCCGTAGATGAATACATTGCTTTTCTCTGATCTATGTATGATGGTACGATCAGAGCTGCTTTTAATTAACATGATCCCTTGCTTTGGCTTGACAAGGATCTAGTCTTATACACAATGGTAAGCCAGTGGTAGTAAAGGTATAAGAAATTTGCTACTATGTTACTGAACCTAGGTGAACGCTAGTATAACTCATTACAAATGTGCTGGAGTAATCAAAGATCGCATTGACGAGCCAACAATGGAAGAGCCAGTCATTTGTCTTGAGACCTATCTAGTTAGTAACTGCTAATGGAACGGTTTCGATATGGATACACAAAAAAAAAAAAAAAAAAAAAAAAAAAAAA

**29. 229E VERAS-3 IFNA14 (5'Leader-TRS-IFN14-3'UTR):**

ACTTAAGTACCTTATCTATCTACAGATAGAAAAGTTGCTTTTTAGACTTTGTGTCTACTTTTCTCAACTGAACGAAAAGATGGCATTGCCCTTTGCTTTAATGATGGCCCTGGTGGTGCTCAGCTGCAAGTCAAGCTGCTCTCTGGGCTGTAATCTGTCTCAAACCCACAGCCTGAATAACAGGAGGACTTTGATGCTCATGGCACAAATGAGGAGAATCTCTCCTTTCTCCTGCCTGAAGGACAGACATGACTTTGAATTTCCCCAGGAGGAATTTGATGGCAACCAGTTCCAGAAAGCTCAAGCCATCTCTGTCCTCCATGAGATGATGCAGCAGACCTTCAATCTCTTCAGCACAAAGAACTCATCTGCTGCTTGGGATGAGACCCTCCTAGAAAAATTCTACATTGAACTTTTCCAGCAAATGAATGACCTGGAAGCCTGTGTGATACAGGAGGTTGGGGTGGAAGAGACTCCCCTGATGAATGAGGACTCCATCCTGGCTGTGAAGAAATACTTCCAAAGAATCACTCTTTATCTGATGGAGAAGAAATACAGCCCTTGTGCCTGGGAGGTTGTCAGAGCAGAAATCATGAGATCCCTCTCTTTTTCAACAAACTTGCAAAAAAGATTAAGGAGGAAGGATTGAACATGCCACTGTGTTGTTTGAAATTCAGGCTTTAGTTGGAATTTTGCTTTTGTTCTTTCTTTTATTATCTTTCTTTTGCCTGTTTTTAGAGAGATTTGGCGCCTTGGTGCCGTAGATGAATACATTGCTTTTCTCTGATCTATGTATGATGGTACGATCAGAGCTGCTTTTAATTAACATGATCCCTTGCTTTGGCTTGACAAGGATCTAGTCTTATACACAATGGTAAGCCAGTGGTAGTAAAGGTATAAGAAATTTGCTACTATGTTACTGAACCTAGGTGAACGCTAGTATAACTCATTACAAATGTGCTGGAGTAATCAAAGATCGCATTGACGAGCCAACAATGGAAGAGCCAGTCATTTGTCTTGAGACCTATCTAGTTAGTAACTGCTAATGGAACGGTTTCGATATGGATACACAAAAAAAAAAAAAAAAAAAAAAAAAAAAAA
